# A Thermochromic, Viscoelastic Nacre-like Nanocomposite for the Smart Thermal Management of Planar Electronics

**DOI:** 10.1007/s40820-023-01149-8

**Published:** 2023-07-05

**Authors:** Jiemin Wang, Tairan Yang, Zequn Wang, Xuhui Sun, Meng An, Dan Liu, Changsheng Zhao, Gang Zhang, Weiwei Lei

**Affiliations:** 1 College of Biomedical Engineering, Sichuan University, Chengdu, 610064 People’s Republic of China; 2 Institute for Frontier Materials, Deakin University, Waurn Ponds Campus, Locked Bag 20000, Geelong, Victoria 3220 Australia; 3 College of Mechanical and Electrical Engineering, Shaanxi University of Science and Technology, Xi’an, 710021 People’s Republic of China; 4 State Key Laboratory of Polymer Materials Engineering, College of Polymer Science and Engineering, Sichuan University, Chengdu, 610065 People’s Republic of China; 5 Institute of High Performance Computing A*STAR, Singapore, 138632 Singapore

**Keywords:** Boron nitride nanosheets, Nacre-inspired composites, Viscoelastic, Thermochromic, Smart thermal management

## Abstract

Cutting-edge heat spreaders for soft and planar electronics require not only high thermal conductivity and a certain degree of flexibility but also remarkable self-adhesion without thermal interface materials, elasticity, arbitrary elongation along with soft devices, and smart properties involving thermal self-healing, thermochromism and so on. Nacre-like composites with excellent in-plane heat dissipation are ideal as heat spreaders for thin and planar electronics. However, the intrinsically poor viscoelasticity, *i.e.,* adhesion and elasticity, prevents them from simultaneous self-adhesion and arbitrary elongation along with current flexible devices as well as incurring high interfacial thermal impedance. In this paper, we propose a soft thermochromic composite (STC) membrane with a layered structure, considerable stretchability, high in-plane thermal conductivity (~ 30 W m^−1^ K^−1^), low thermal contact resistance (~ 12 mm^2^ K W^−1^, 4–5 times lower than that of silver paste), strong yet sustainable adhesion forces (~ 4607 J m^−2^, 2220 J m^−2^ greater than that of epoxy paste) and self-healing efficiency. As a self-adhesive heat spreader, it implements efficient cooling of various soft electronics with a temperature drop of 20 °C than the polyimide case. In addition to its self-healing function, the chameleon-like behavior of STC facilitates temperature monitoring by the naked eye, hence enabling smart thermal management.

**Supplementary Information:**

The online version contains supplementary material available at 10.1007/s40820-023-01149-8.

## Highlights

- Construction of a viscoelastic composite nacre with a ripple-like layered architecture through supramolecular interactions.
- Outstanding self-adhesion, self-healing and scrape-resistant mechanical and thermal properties.
- Utility as an integrated heat spreader and TIMs for “chameleon-like” thermal management of planar soft electronics.

**Supplementary Information:**

The online version contains supplementary material available at 10.1007/s40820-023-01149-8.

## Graphical abstract

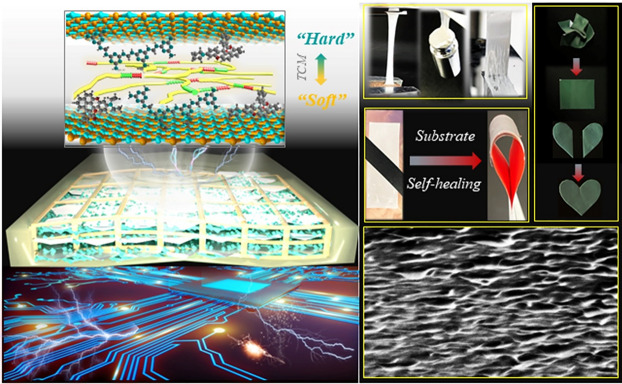

## Introduction

Nacre-inspired composites with layered architecture have attracted extensive attention due to their specific anisotropy, high strength and strong practicability in engineering-related domains [1–5]. Especially for use in heat dissipation, the lamellar structure promotes excellent phonon transportation along the radial direction, enabling fast in-plane heat spreading [6–8] In contrast with other heat conductors, this unique structural feature provides overwhelming advantages in horizontal heat dissipation, making it perfect for miniaturized, thin and planar devices. Since the first report of artificial nacre-like composite paper for light-emitting diode (LED) chip cooling in 2015 [9], the past few years have witnessed an increase in studies on nacre-biomimetic composites in thermal management applications. These composite materials normally consist of two-dimensional (2D) thermoconductive fillers, such as graphene [7, 10], boron nitride nanosheets (BNNS) [4, 5, 11] and MXene [12], and polymeric matrices, including polyvinyl alcohol (PVA) [9, 13] poly dimethyl diallyl ammonium chloride (PDDA) [14], polyimide (PI) [15] polyvinylidene fluoride (PVDF), [16] epoxy [17] cellulose nanofibers (CNF) [18] and aramid nanofibers (ANF) [19, 20]. Although many breakthroughs have occurred in the enhancement of in-plane thermal conductivity (ҡ_//_) as well as flexibility and ductility, these factors remain critical for integration with soft electronics. First, almost all thermoconductive artificial nacres lack sufficient adhesiveness. Thus, instead of self-sticking, they rely on silver paste or epoxy adhesives as thermal interface materials (TIMs) to attach devices for cooling, which inevitably results in interfacial mismatch, loss of ҡ_//_ and glue peel-off under long service life. In addition, the majority of composites with nacreous architecture possess high stiffness and lack stretchability and elasticity, preventing arbitrary elongation together with current flexible electronics such as bioimpedance tattoos and e-skin [21, 22]. In fact, it seems contradictory to combine intrinsically tough nacre and high viscoelasticity in a single material [23], which does not exist in nature. However, this is also an opportunity for novel nacre-like composite designs to circumvent the usual “hard/soft” trade-off, making them attractive choices for such applications.

In addition, unlike conventional chips or transistors that are lie a machine, the circuits and displays in planar electronics are usually exposed on the body surface for transparency and visibility [24, 25]. Thus, the naked-eye embodiment of smart technologies is in high demand in modern thermal management systems, particularly for flexible devices [26]. In addition to traditional properties such as high thermal conductance, light weight and deformability, advanced heat dissipating materials have also been developed with multiple functions including self-healing ability, thermal responsiveness and temperature sensing ability [27–29]. Nevertheless, combining these attributes into a nacre-like composite is a difficult task: the internal layered texture impedes the mobility of encapsulated agents for extrinsic self-repair [27], and the rigid fillers show poor compatibility with the soft polymer matrix, which increases the interfacial thermal resistance and limits the movement of polymer chains for rapid thermal response [28]. To mediate the intramolecular interactions among multiple components, existing work often introduces extra molecular moieties to construct H-bonding networks or conjugation systems between fillers and polymeric matrices [30–33]. However, improving the thermal conductivity without compromising other functions of the composite is still challenging.

Herein, we design a soft thermochromic composite (STC) with both a nacre-like structure and viscoelasticity by rationally assembling water-dispersible self-healing polyurethane (W-SPU), functionalized BNNS and thermochromic molecules (TCMs) through aqueous vacuum filtration (VAF). Both theoretical and experimental analyses certified the molecular engineering effect of TCMs for the enhancement of adsorption energy and overlap in phonon density of states between “hard” BNNS and “soft” W-SPU, thus comprehensively improving the mechanical and thermal performances. The resultant composite membrane conveyed a high ҡ_//_ of ~ 30 W m^−1^ K^−1^, a low thermal contact resistance (*R*_c_) of ~ 12 mm^2^ K W^−1^ and a large elongation of ~ 500%. In addition to those fundamental properties, the STC nacre exhibited remarkable adhesion with different substrates, fast self-healing and steady thermochromic behaviour. As a proof-of-concept, our work validated the practicability of smart thermal management for planar electronics and circuits. Additionally, STC nacre shows great potential in future applications such as thermal biomimetics, thermal biosensors and soft robots.

## Experimental Section

### Materials

h-BN powders were purchased from Momentive Performance Materials, Inc. TCM was purchased from Insilico, Inc. Commercial TIM grease (ҡ ~ 4 W m^−1^ K^−1^, X-23–7762) was purchased from Shin-Etsu Chemical Co., Ltd. Commercial silicon TIM pads (ҡ ~ 5 W m^−1^ K^−1^) were purchased from Dongguan Sheen Electronic Technology Co., Ltd. Epoxy adhesives were purchased from Shanghai Shouxing Industrial Co., Ltd. A commercial aluminium (Al) foil Kirigami electrode with a polyimide substrate (30 mm × 35 mm, 12 V, 5 W) was purchased from Fangzhou thermoelectric and electronic Co., Ltd. Other chemicals were purchased from Sigma‒Aldrich. All reagents were used as received without purification.

### Preparation of BNNS, W-SPU and STC Membranes

#### Preparation of the BNNS

h-BN powders and urea were mixed and milled through a ball milling machine (Pulverisette 7, Fritsch), followed by dialysis in water. The details can be found in our previous research [34].

#### Preparation of the W-SPU Aqueous Dispersion

Ten grams of PTMEG were heated at 110 °C under vacuum overnight to remove moisture. Then, the PTMEG reacted with 7 g of 4,4’-MDI and 1 g 2,2-Bis(hydroxymethyl)propionic (DMPA) in 100 ml DMF in the presence of 0.04 g DBTDL under N2 at 70 °C. Next, 2 g of HEDS was added to the mixture as a chain extender. After completion of the reaction, the temperature was decreased to 30 °C, and acetone was added to reduce the viscosity of the system. Afterwards, 0.8 g TEA was added for acid-alkaline neutralization. Finally, 50 mL of DI water was added to form the W-SPU emulsion, followed by agitation at 2000 rpm.

#### Preparation of the STC-2 Membranes

For this purpose, 10 mL of BNNS aqueous solution (3 mg mL^−1^) was first mixed with 540 µL of W-SPU solution (130 mg mL^−1^). The aqueous mixture was diluted to 100 mL with DI water. Then, 1 mL of TCM ethanol dispersion (3 mg mL^−1^) was added to the diluted liquid. After stirring for 1 h, the mixture solution was filtered under vacuum. The as-achieved membrane could be slightly peeled off from the separator when it is not fully dried. We kept it in a PTFE box. Notably, long-term storage may cause adhesion of the membrane on the substrate. Other STC membranes were fabricated similarly except for the different contents of BNNS.

### Material Characterization

X-ray diffraction (XRD) patterns were obtained on a PANalytical X’Pert apparatus with Cu Kα radiation. Small-angle X-ray scattering (SAXS) data were obtained from a Xeuss 2.0. An incident X-ray beam with an energy of 15 keV was used. XPS analysis was collected on a Kratos AXIS Nova instrument with Al K*α* X-rays as the excitation source. Fourier transform infrared (FTIR) spectra were obtained via a Nicolet 7199 FTIR spectrometer. Temperature-dependent FTIR spectra were obtained from a Nicolet iS50 Fourier transform spectrometer. The membranes were heated from 25 to 75 °C at 5 °C min^−1^. 2D correlation results were calculated by using the software 2D Shige ver. 1.3 (©Shigeaki Morita, Kwansei-Gakuin University, Japan, 2004–2005). The contour maps were drawn by the Origin 2018 program. In the contour plots, a warm colour (red) represents positive intensities, whereas a cold colour (blue) represents negative intensities. Thermogravimetric analysis (TGA) was performed on a TA Instruments Q50 at 10 °C min^−1^ from 25 to 800 °C under air flow. Dynamic mechanical analysis (DMA) tests were conducted in a Metravib 450 + from 25 to 100 °C at 5 °C min^−1^ under 1 Hz. ҡ was tested with an LFA 457 analyser (NETZSCH, Germany) and calculated according to the equation *ҡ* = *α* × *ρ* × *C*, where *α*, *ρ*, and *C* represent the thermal diffusivity, density, and specific heat capacity, respectively. The density was calculated by a weighing method. The specific heat capacity was measured by a differential scanning calorimeter (Q2000, TA Corporation, USA). The surface temperature was recorded by an infrared thermograph (FILR T560). The thermal contact resistance was measured through a TIM-tester (ASTM D5470) with an aluminium (Al) plate as the end material. The thermal expansion value was recorded by a DIL L75. Scanning electron microscopy (SEM) and transmission electron microscopy (TEM) imaging were executed on a Zeiss Supra 55 VP instrument and a JEOL 2100F microscope operating at 200 kV, respectively. Atomic force microscopy (AFM) was performed on a Cypher atomic force microscope. The zeta potential was measured by a Zetasizer Nano ZS90 apparatus from Malvern. The mechanical measurements were tested by an Instron 30 KN tensile tester on a 50 N load cell with a loading rate of 10 mm min^−1^. The adhesion force was tested by lap shear methods, and the cyclability of adhesion was tested by compression mode in Instron. The scarping test was conducted by an HSR-M wearing machine.

### Simulation

The computational methodology for the detailed simulation is provided in the supporting information.

## Results and Discussion

### Design Principle and Structural Characterizations

**Fig. 1:**
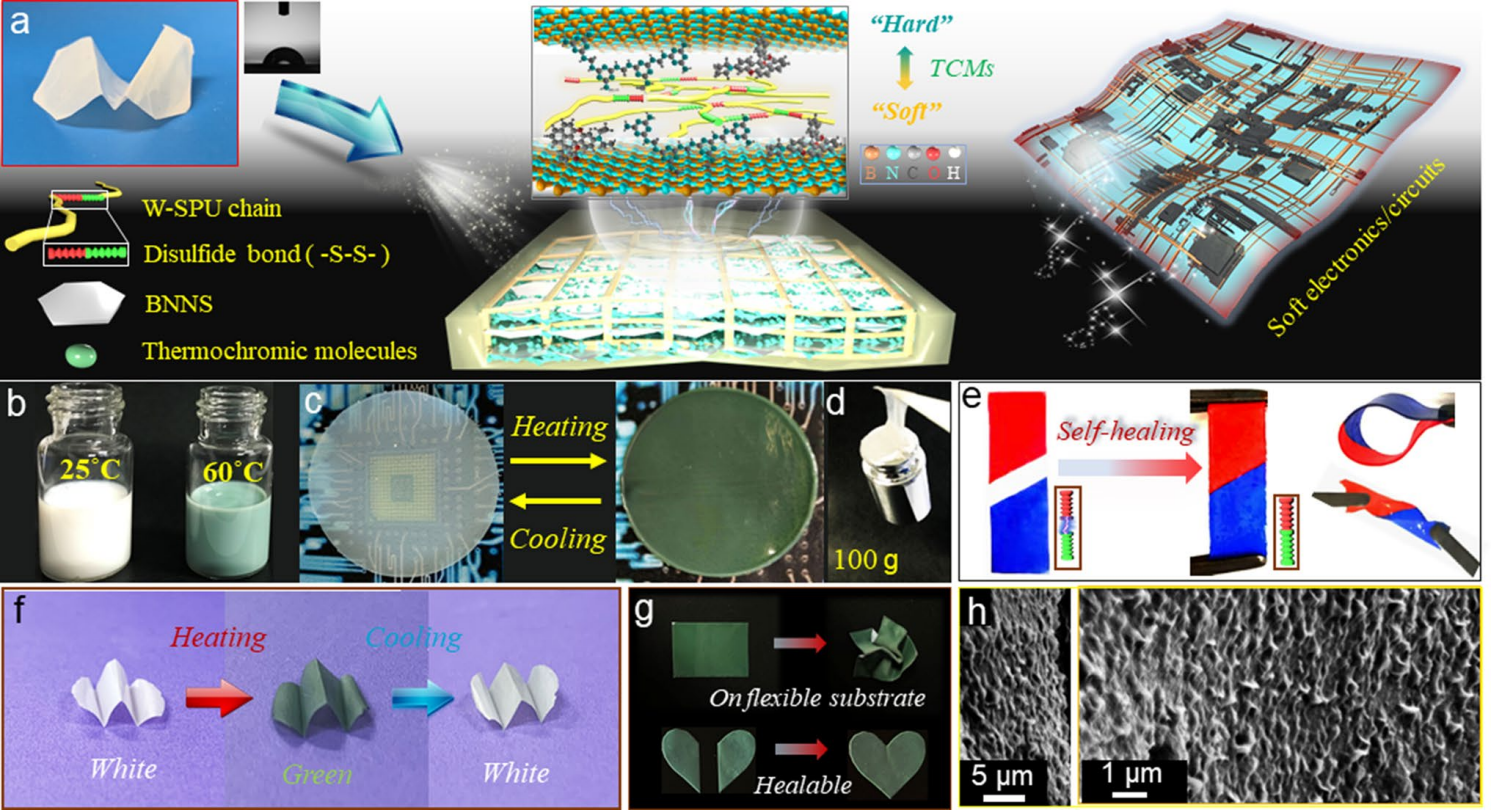
**a** Photos and schematic images of the STC membrane composed of W-SPU, BNNS and TCMs via the VAF strategy and its corresponding application for planar electronics/circuits. The inset displays the flexibility and contact angle of the STC membrane. **b** Photo of the thermochromic STC inks. **c** Photos of the STC membranes with thermochromic properties. **d** Photo of the STC membrane showing the self-adhesive tensile stress. **e** Photos of STC membranes showing the self-healing property (the cut slices dyed red and blue). **f** Photos showing the stable colour change of the STC-2 membrane during folding. **g** Photos showing the outstanding thermochromic performance of the STC membrane self-adhered to a soft substrate (top) and cut for self-healing (bottom). **h** SEM images of the cross-section of the TIM composite membrane

The design of STCs through a rational assembly of ternary moieties is shown in Fig. 1a. To enable both self-adhesive and self-healing properties, we initially synthesized polyurethane with disulfide bonds (SPU) as the polymer matrix (Fig. S1). The dynamic disulfide bond in the SPU chain softened the polymer backbone, enhancing the mobility and thus increasing the viscidity and adhesiveness (Fig. S2) [35, 36]. Then, amino-group functionalized BNNS with good water dispersibility was used as a highly thermoconductive filler. The successful synthesis of BNNS according to previous work [34] has been confirmed in Figs. S3-S6. To enhance the compatibility of SPU and BNNS in aqueous solution, we further utilized 2,2-bis(hydroxymethyl)propionic acid (DMPA) and triethylamine (TEA) to modify SPU to waterborne SPU (W-SPU) [33] aiming to furnish water solubility (contact angle ~ 60°) (Fig. 1a inset and Fig. S7). Afterwards, an ethanol dispersion of TCM comprising urea–formaldehyde resin and 6’-diethylamino-1’,2’-benzofluoran was added (Fig. S8). The addition of the third component not only provided thermochromic properties but also improved the interfacial miscibility between soft W-SPU chains and hard BNNS segments via molecular engineering. As shown in Fig. 1b, the aqueous ink mixture of W-SPU, BNNS and TCM appeared milk-white at room temperature, while the colour changed to green when heated above 60 °C. The phenomenon reflected good homogeneity and heat-stimulus responsiveness of the ink. Furthermore, nacre-like STC membranes can be easily achieved via VAF fabrication from a mixed water solution. As demonstrated in Figs. 1c and S9, the membrane inherited the colour-changeable feature upon heating and cooling. Moreover, when we directly attached it to a 100 g weight, it was able to lift the weight by sticking to the surface (Fig. 1d), indicating strong self-adhesiveness. In addition, excellent self-healing performance is illustrated in Fig. 1e. After cutting and healing, the membrane was able to undergo arbitrary bending and twisting. Figures 1f, g and S10 verify consistent thermochromic performance even under folding, self-adhering on flexible substrates, cutting for self-healing, or scraping for recovery. Hence, it reflected promising viscoelastic properties integrating flexibility, self-adhesive/self-healing and thermochromic properties. Then, scanning electron microscopy (SEM) clearly revealed a nacreous architecture of the STC membrane in the cross-section (Figs. 1h and S11). Interestingly, this morphology showed a more compact and rippled lamella than other reported nacre-like composite membranes with straight and brick-like lamellar structures [13–15]. This may stem from the strong adhesiveness of W-SPU, which tightly binds adjacent layers. This layered structure is thus believed to grant outstanding ҡ_//_ for STC.

**Fig. 2:**
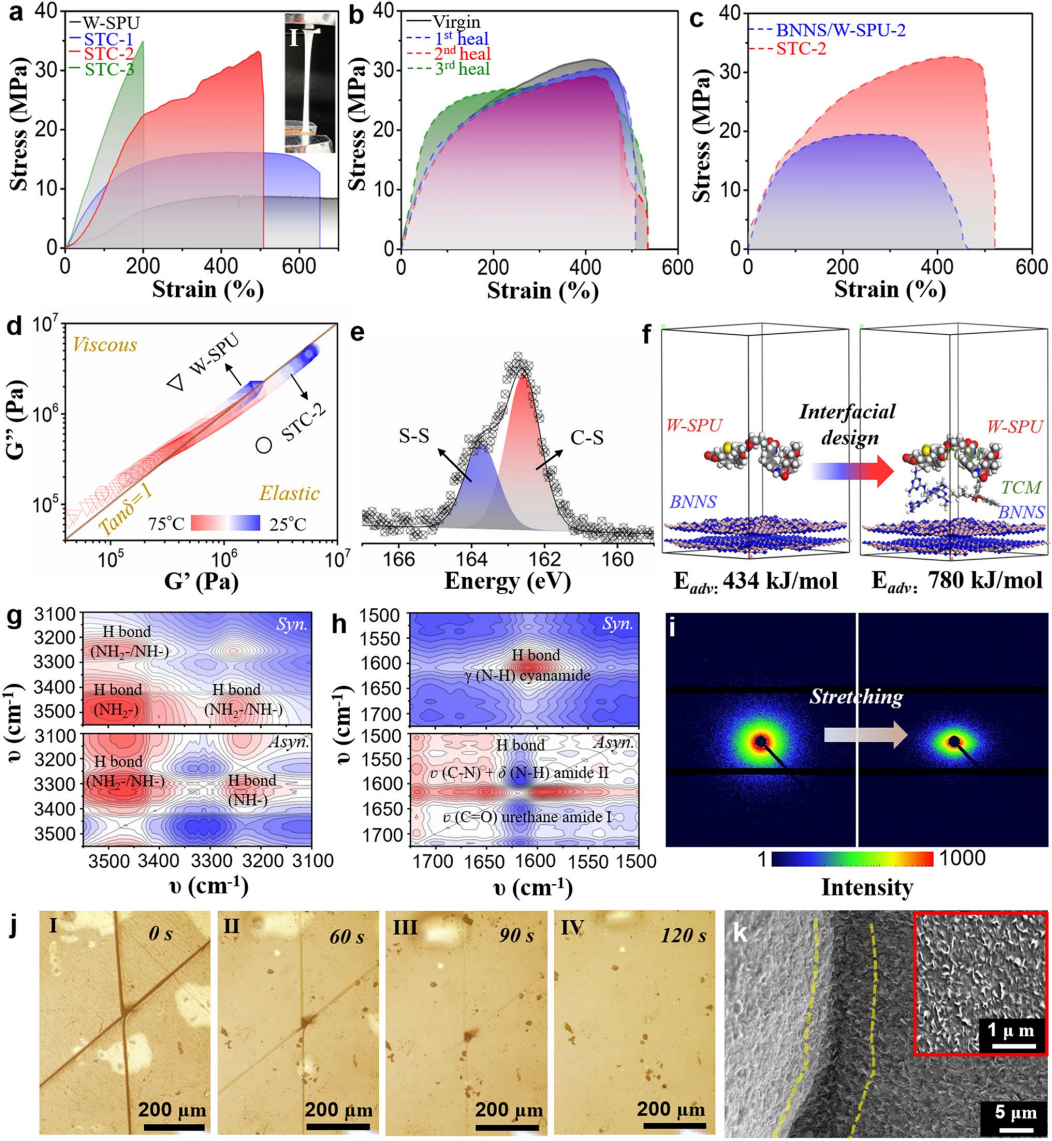
**a** Tensile stress of different STC membranes (I: Photo shows the elongation of STC-2.). **b** Tensile stresses and self-healing performances for STC-2. **c** Comparison of tensile stress between STC-2 and W-SPU/BNNS-2. **d** DMA of W-SPU and STC-2. **e** XPS of S in the STC after self-healing. **f** Optimized structure simulation and molecular adsorption energy calculation for STC and W-SPU/BNNS. **g** 2D COS synchronous and asynchronous spectra of STC from 3550 to 3100 cm^−1^. **h** 2D COS synchronous and asynchronous spectrum of STC from 1725 to 1500 cm^−1^. (The warm colour (red) represents positive intensities, while cold colours (blue) represent negative intensities). **i** SAXS images of STC-2 before and after uniaxial stretching to 300%. **j** Optical images of the scratch self-healing process of STC-2. **k** SEM images of the scratch after self-healing of STC-2. The magnified SEM image highlights the surface area of the intact nanosheet network

We then tailored the STC membranes with various BNNS contents and fixed TCM content, naming them STC-1 (~ 15–18 wt% BNNS), STC-2 (~ 30–32 wt% BNNS) and STC-3 (~ 63–65 wt% BNNS). The weight fractions were quantitatively confirmed by thermogravimetric analysis (TGA) (Fig. S12). The mechanical performances are illustrated in Fig. 2a. The original W-SPU possessing high viscoelasticity was a transparent elastomer and could be stretched to ~ 2000% strain (Fig. S13a). However, the tensile stress was relatively low (~ 8 MPa), especially after modification with SPU. The introduction of BNNS resulted in a larger modulus above 20 MPa since the BNNS owned rigidity. Under ~ 30% wt of BNNS loading, the break elongation of STC-2 reached approximately 500% (Fig. 2a-I and S13), which is superior to that of other reported VAF-derived nacre-like composite membranes (*i.e.,* BNNS/PVA [9, 13], BNNS/PI [15] and BNNS/CNF [18], BNNS/ANF [19, 20], etc.). Here, we chose STC-2 as the optimal case by overall consideration of the mechanical, self-adhesive/self-healing and thermal properties. Impressively, the ultimate self-healing efficiency can reach approximately 85%, even after 3 cycles of complete fracture and recovery (Fig. 2b). The effect of TCM on mechanical reinforcement is illustrated in Fig. 2c. Noticeably, both the tensile stress and elongation rate were increased by 50% and 16%, respectively. From the chemical structures, the TCM contributed to interactions with both W-SPU and BNNS via H-bonding and π-π stacking (Fig. S8), hence benefiting the increment [37]. To be more specific, we adopted dynamic mechanical analysis (DMA) to investigate the mechanical changes alongside the temperatures. In Figs. 2d and S14, the dependent curves of storage modulus G’ and loss modulus G’’ with loss factor (tan*δ*) are displayed. At room temperature, W-SPU was viscous, while both BNNS/W-SPU-2 and STC-2 preferred viscoelasticity. The G’ and G’’ for STC-2 were increased by 209% and 148%, respectively, which were markedly greater than those of BNNS/W-SPU-2: G’ by 48% and G’’ by 30%. On the one hand, this can be ascribed to the formation of physical crosslinking points by BNNS that restrained the mobility of soft polymer chain segments, thereby enhancing the toughness accordingly. On the other hand, differential scanning calorimetry (DSC) curves revealed that STC-2 had a higher glass transition temperature than the others (Fig. S15). Compared to the original W-SPU with an amorphous phase, there were endothermic peaks for TCM crystallites of STC-2, which was consistent with the XRD results (Fig. S16). Thus, in addition to H-bonding and π-π stacking effects, the small TCM crystallites also imposed interactions on polymer chain movement, therefore facilitating viscoelasticity. To exclude the possibility of BNNS and TCM impacting dynamic disulfide reconnection, the sulfur element constitution of STC-2 was measured after heating and cooling by X-ray photoelectron spectroscopy (XPS) (Fig. 2e). The peaks at approximately 162.4 and 163.8 eV originated from the C‒S and S‒S bonding in the polymer backbone [38]. No dissociative S- or S‒O bonding was detected, implying that the self-adhesive/self-healing mechanism based on dynamic disulfide was not damaged. We further simulated the adsorption energies between the W-SPU chain and BNNS layer (with or without TCM) to theoretically prove the intramolecular interactions (Figs. 2f and S17) [39]. The adsorption energies were calculated after optimizing the molecular structures and conformation stabilization (Table S1). The outcome showed that the employment of TCM increased the adsorption energy to 780 kJ mol^−1^ in the system, ~ 80% improvement over the energy between W-SPU and BNNS (434 kJ mol^−1^). Further investigation of the interactions was performed by temperature-dependent FTIR spectra over a range of 25–75 °C. There were intensive overlapping representative peaks in the ranges of 3600–3000 and 1800–1500 cm^−1^, mainly assigned to the H-bonds of N–H, *ʋ* (C = O) urethane amide and *ʋ* (C = N) triazine from W-SPU and cyanamide in TCM (Fig. S18). In the 2D correlation spectroscopy (COS) spectra from 3550 to 3100 cm^−1^ (Fig. 2g), a strong cross band with positive intensity at 3480 cm^−1^ for H-bonding of NH_2_– and a cross band with negative intensity at 3325 cm^−1^ for H-bonding of–NH- were identified. No correlated band can be assigned in the asynchronous spectrum. The results implied that the interfacial interaction between BNNS and TCM was improved by forming stronger H-bonding between each NH_2_- group [27]. Similarly, in the 2D COS spectra from 1725 to 1500 cm^−1^ (Fig. 2h), the cross band with a positive intensity at 1620 cm^−1^ corresponded to H-bond accumulation with *γ* (N–H) cyanamide in TCM. In contrast, both the H-bonds of *ʋ* (C = O) urethane amide I at 1670 cm^−1^ and *ʋ* (C–N) + *δ* (N–H) amide II at 1520 cm^−1^ were synchronously weakened [40]. This result reflected that TCM indeed enabled reinforcement by the formation of supramolecular interactions via H-bonding upon heating, exactly corresponding to the DMA analysis. Furthermore, we employed SAXS to probe the positive effect of TCM on the alignment of the BNNS network in the W-SPU matrix during stretching. The original W-SPU membrane presented no obvious scatter signals before and after drawing (Fig. S19a). For viscoelastic STC-2, the 2D scattering pattern ring became narrow and rhombic-like from a pristine circle under elongation to 300% strain (Fig. 2i), symbolizing the orientation of the BNNS network [34]. For the composite lacking TCM, the degree of orientation was clearly lower simply with an ellipse ring (Fig. S19b). The outcome can explain the rational design of ternary nacre-like composites by incorporating TCM, not only for the thermochromic response but also for engineering the interface at the molecular level. The optical images displayed the dynamic healing process of scratching in the STC-2 membrane (Fig. 2j). The crack fully disappeared after 120 s at 70 °C. To be more precise, we carefully checked the surface area after healing through SEM images (Fig. 2h). The subtle trail representing successful recovery was detected under magnified SEM. Appreciably, the BNNS network with densely aligned nanosheets remained intact throughout the W-SPU matrix, thus being able to heal invisible internal damages.

### Mechanical and Thermal Performances of Self-adapting Substrates

**Fig. 3:**
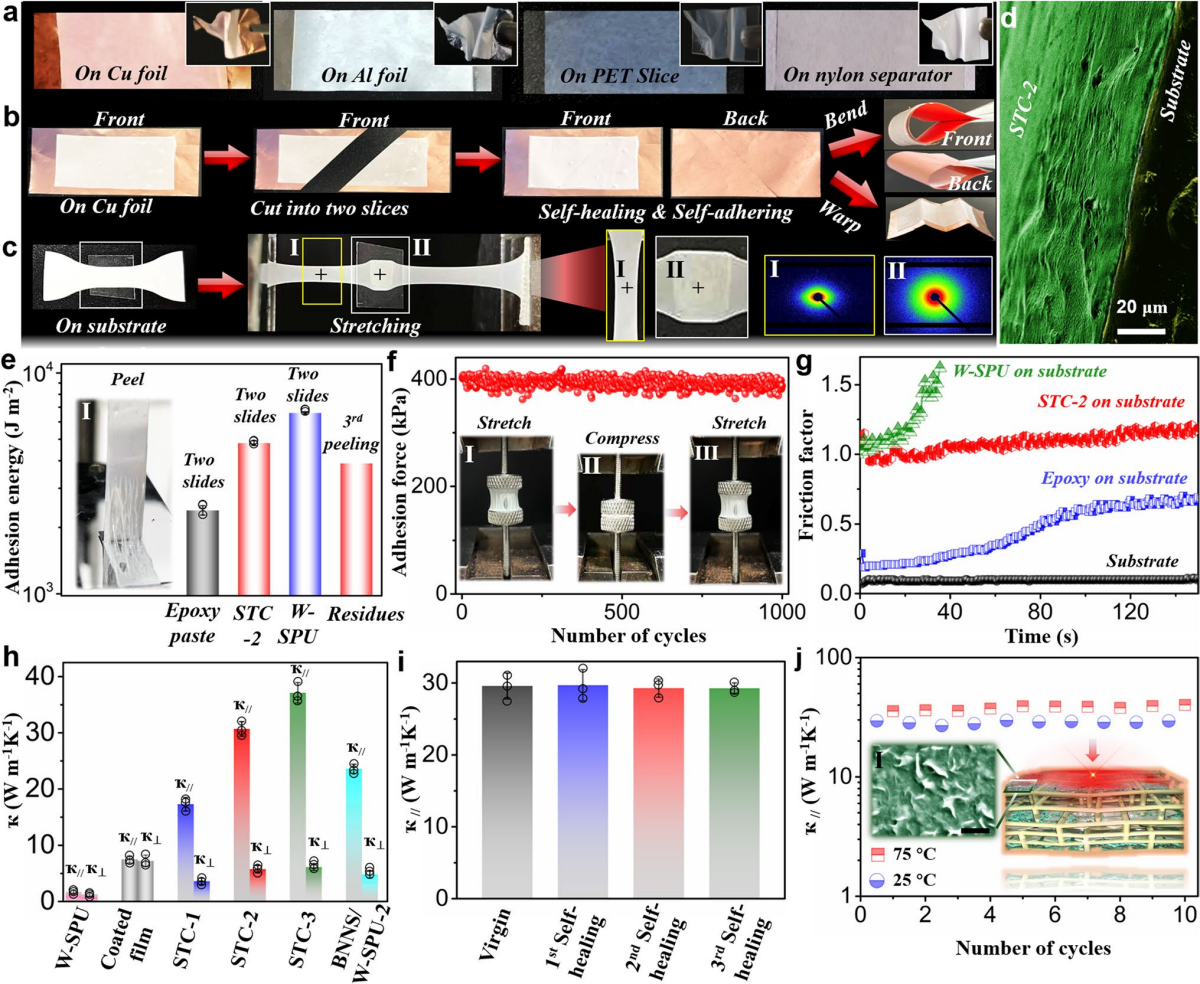
**a** Photos showing the excellent self-adhesiveness of the STC-2 membrane on various substrates. **b** Photos showing the repair of substrate by self-adhering STC-2 membrane. **c** Photos and SAXS images to demonstrate the excellent interfacial adhesiveness after stretching. **d** SEM image of the interface between the self-adhering STC-2 membrane and the substrate. **e** Adhesion energy of epoxy paste, STC-2, W-SPU and residues from the 3.^rd^ peeling (I: Photo shows the peeling of STC-2 from the substrate.). **f** Adhesion force of STC-2 after 1000 cycles of contact and separation (I-III: photos of the test process). **g** Friction factors of different samples. **h** ҡ of different samples. **i** ҡ_//_ of STC-2 after self-healing. **j** Temperature cyclability of ҡ_//_ from 25° to 70° (I: SEM image of BNNS at the surface area, scale bar: 200 nm)

Then, the self-adhesive property of the nacreous STC-2 membrane was estimated, as illustrated in Fig. 3a. It can be directly attached to various flexible substrates involving Cu foil, Al foil, PET slices and nylon separators. Through rolling, twisting and deforming, it remained firmly to those substrates without peeling off (Video S1). The impressive self-adhesiveness thereby showed versatility for thin, soft and flexible electronics. Even if cut into two pieces, the STC-2 self-adhered Cu foil could be spliced by joining those segments (Fig. 3b and Video S2). To deeply manifest the considerable interfacial self-adhesion, we applied SAXS to detect the structural changes of the membrane adhering to the substrate. As displayed in Fig. 3c, after stretching, both ends not bound to the substrate were elongated to a slim stripe, whereas the middle part attached to the substrate almost retained its original dimensions. Through SAXS analysis by picking positions I and II, the area without adhesion showed a narrow rhombic-like pattern due to the change in orientation by elongation. In contrast, despite tension, the middle area reflecting the dispersion circle ring represented no change in orientation (Fig. S20). It thus proved a strong interfacial adhesion that can resist bilateral strain. The morphology of the interface between STC-2 and the substrate was characterized by SEM. Evidently, the structurally nacre-like membrane adhered tightly to the substrate without leaving any gaps or voids.

**Fig. 4:**
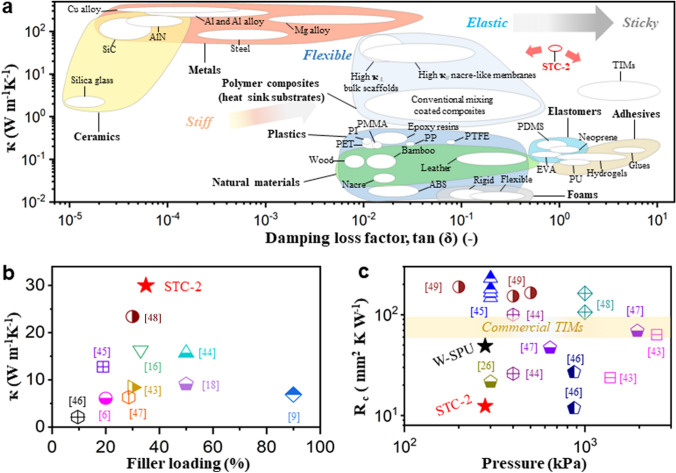
**a** Comparison of ҡ and damping loss factor among different materials. **b** Comparison of ҡ among currently reported polymer composites. **c** Comparison of *R*_c_ and packing pressure of STC-2 with various TIMs

The adhesion energy was calculated from two slides shearing (Figs. 3e and S21). Pristine W-SPU possessed a high adhesion energy of 6584 J m^−2^. For nacre-like STC-2, although the adhesion energy reasonably decreased, it still reached 4607 J m^−2^ for STC-2, greater than 2387 J m^−2^ of commercial epoxy paste and other reported adhesives [41]. Moreover, the self-adhered sticky residues attained 81% adhesion energy after 3 peeling cycles, highlighting their outstanding adhesive ability (Fig. S22). The inset photo I in Fig. 3e intuitively displays the adhesive power when peeling STC-2 from the substrate. Good cyclability of adhesion was also observed under repeated touching and peeling (Fig. 3f and inset photos I-III). After 1000 cycles, 96% of the adhesion force was retained at ~ 400 kPa, highlighting the long-term durability as adhesives. The surface friction factors of pure plastic polyethylene (PE), epoxy paste-adhered PE, STC-2-adhered PE and W-SPU-adhered PE were then measured. As shown in Fig. 3g, pure W-SPU was so cohesive with high viscosity that it delivered the highest friction factor. However, it was too soft to withstand the striking of the swinging pin from the wearing machine. For STC-2, the nacre-like layered network effectively offered a certain toughness as well as lubrication, hence protecting it from stabbing. The good anti-scraping property due to the inherent viscoelasticity of the composites was beneficial to prevent scratch-induced thermal conductance loss. The thermal conductive performances are subsequently illustrated in Fig. 3h. STC-2 conveyed a ҡ_//_ of ~ 30 W m^−1^ K^−1^ and ҡ_⊥_ of ~ 5.8 W m^−1^ K^−1^. The large thermal anisotropy was reasonable because of the nacre-like laminar structure (Fig. S23) [13–15]. By comparison, the drop-coated composite membrane simply showed a ҡ_//_ of ~ 7.5 W m^−1^ K^−1^ and ҡ_⊥_ of ~ 6.8 W m^−1^ K^−1^ due to the random distribution of BNNS in the polymer matrix. More impressively, adding TCM for STC-2 allowed the enhancement of *ҡ*_//_ by 35%. *ҡ*_//_ remained invariable during 3 cycles of self-healing, justifying the good recoverability (Fig. 3i). We continued testing ҡ_//_ at 25 and 75 °C separately and found it to be temperature-dependent (Fig. 3j). At 75 °C, ҡ_//_ grew to ~ 40 W m^−1^ K^−1^, owing to the elevated viscosity and liquidity that enabled more intensive contact between adjacent BNNS on the surface of the layer (Fig. 3j inset) [42]. In fact, the density of nanosheets in STC can be rationally tuneable to manipulate the contacts (Fig. S24). To better define STC-2, ҡ versus tan*δ* was compared among multitudinous materials in Fig. 4a [23]. For ceramics and metal materials as heat sinks, in spite of ultrahigh ҡ, the foldability and stretchability were limited due to the relatively high stiffness and brittleness, whereas natural materials and polymers possessing low *ҡ* were not competent to dissipate heat. We then compared the ҡ of STC-2 with those of other polymer composites, such as BNNS/CNF membranes, polypropylene/r-GO membranes, and MXene/PU films (Fig. 4b) [6, 9, 16, 18, 43–48]. Clearly, STC-2 possessed the highest ҡ value at a relatively low filler loading, verifying its excellent thermal conductance. Moreover, although recently developed polymer composites involving artificial nacres had a large *ҡ*_**,**_ regardless of whether they were in-plane or out-of-plane, they were not intrinsically adhesive, inevitably requiring TIMs as interlayers, resulting in a lack of mechanical and thermal interfacial adaptability. However, the gluey TIM fluids with comparatively lower ҡ and poor rebound resilience hardly enabled application as direct heat-sink substrates for soft electronics. In our case, combining high ҡ_//_, flexibility and viscoelasticity, STC-2 can serve as an integrated heat spreader and TIM. Of course, the low thermal contact resistance (*R*_c_) value is also necessary for attaching STC-2 directly to flexible electronics rather than applying extra TIM paste. From the *R*_c_ measurement in Fig. 4c, the *R*_c_ (12.4 mm^2^ K W^−1^ under 280 kPa) of STC-2 (thickness: ~ 50 μm) was much lower than that of commercial TIMs (60–80 mm^2^ K W^−1^) and most reported TIMs that were tested at higher packing pressures [26, 43–49], affirming the reliability.

### Theoretical Simulations of Interfacial Thermal Resistances

**Fig. 5:**
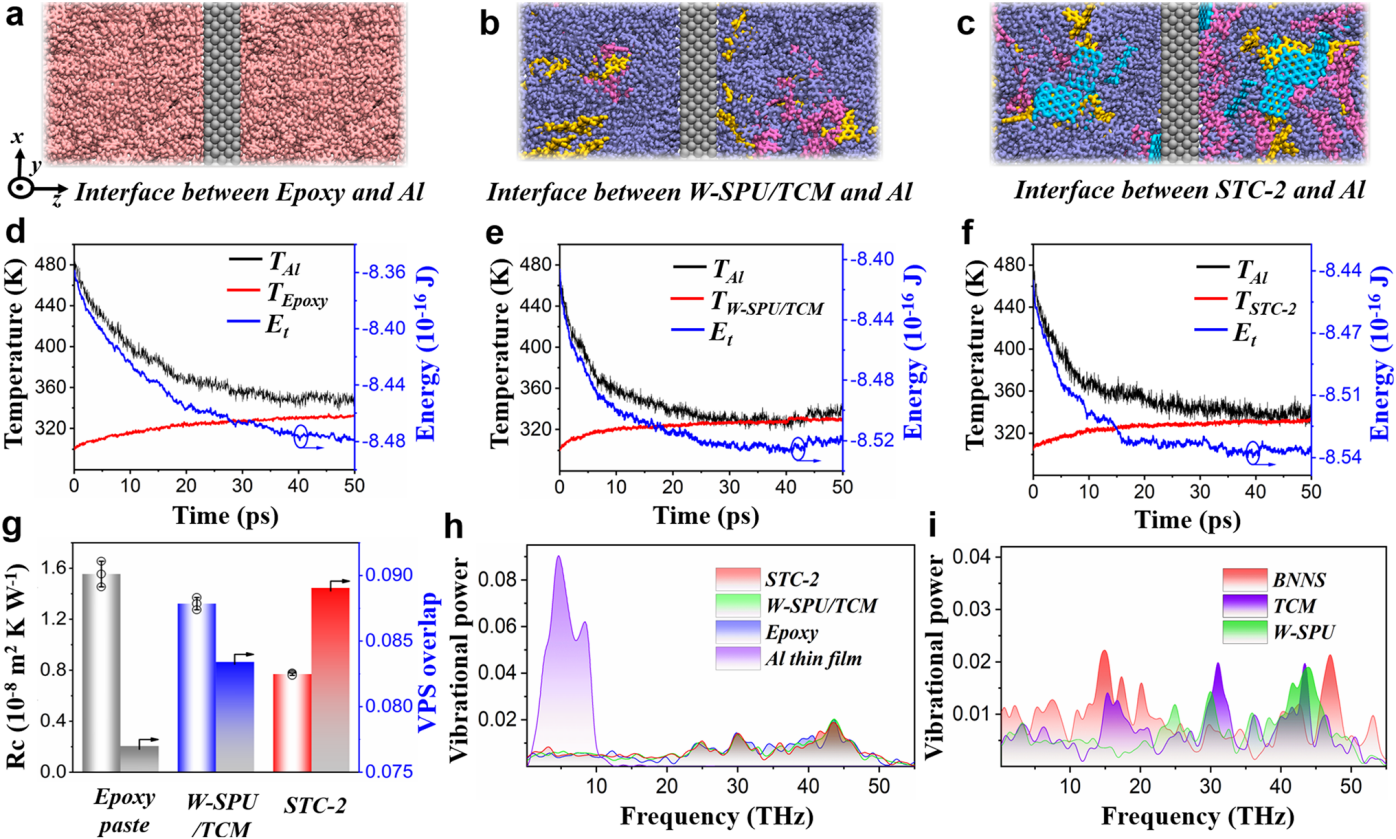
Molecular dynamics simulation systems of interfacial thermal resistances where a thin Al film is embedded in **a** epoxy adhesive (pink colour), **b** W-SPU/TCM (blue colour denotes W-SPU, yellow colour and mauve colour denote the TCM molecules), **c** STC-2 (cyan colour denotes the BNNS). **d-f** Temperature and energy evolutions of the Al thin film and three polymer systems. **g** Interfacial thermal resistances between the Al thin film and epoxy paste, W-SPU/TCM, STC-2. The overlap of the VPS of two materials at these two interfaces. **h** VPS of materials in three types of interface systems. **i** VPS of each component in the STC-2 system

The *R*_c_ and relatively promising interfacial thermal properties of STC-2 in addition to the nacre-like structure were further elucidated theoretically by molecular dynamics (MD) simulation. We built models of interfacial thermal resistances between Al film and commercial epoxy adhesives, W-SPU/TCM, and STC-2 (Fig. 5a-c). The temperature and energy evolution profiles suggested that both W-SPU/TCM and STC-2 displayed greater heat conduction than the epoxy (Fig. 5d-f) [50]. The calculated interfacial thermal resistance values between the Al film and commercial epoxy paste, W-SPU/TCM, and STC-2 were 1.59 × 10^−8^, 1.32 × 10^−8^, and 0.72 × 10^−8^ m^2^ K W^−1^, respectively (Fig. 5g left), conforming to the experimentally observed trends in Fig. 3e. Additionally, to reveal the underlying microcosmic thermal transport mechanism at the interface, the modelled vibrational power spectra (VPSs) of the Al thin film, W-SPU/TCM, STC-2 and epoxy paste were calculated, as shown in Fig. 4h. For epoxy adhesives, W-SPU/TCM and STC-2, the VPSs showed a broad frequency distribution from 0 to 55 THz, whereas it only appeared in the low-frequency range (< 10 THz) for the Al film, ascribed to the intrinsic phonon transport nature among different materials, *i.e.,* metals, organic molecules or polymers. To quantify the overlap of the VPS at these two interfaces (Al thin film and other systems), the cosine similarity measure was applied as follows [51]:

1 $$ S = \frac{{\smallint V_{A} \left( \omega \right) \cdot V_{p} \left( \omega \right){\text{d}}\omega }}{{\left( {\smallint V_{A} \left( \omega \right)^{2} {\text{d}}\omega } \right)^{1/2} \cdot \left( {\smallint V_{p} \left( \omega \right)^{2} {\text{d}}\omega } \right)^{1/2} }} $$

where *S* is the value of similarity, and *V*_*A*_ (ω) and *V*_*p*_ (*ω*) are the VPSs of the Al and polymer systems at the interface, respectively. As predicted, the obtained values in Fig. 5g (right) indicated that Al-STC-2 possessed the maximum overlap with the optimal phonon match between interfaces, followed by Al-W-SPU and Al-epoxy. Additionally, in the ternary component system of STC-2, we found that the VPS peaks of TCM broadly overlapped in the low-frequency range with BNNS and in the high-frequency range with W-SPU (Fig. 5i). That is, it can act as a bridge to connect those two incompatible phases to promote phonon transfer. The phenomenon therefore demonstrated the contribution of small molecular TCM for promoting interfacial compatibility and heat transfer, in accordance with the above experimental characterizations.

### Practical Applications for Thermal Management of Soft Planar Electronics

**Fig. 6:**
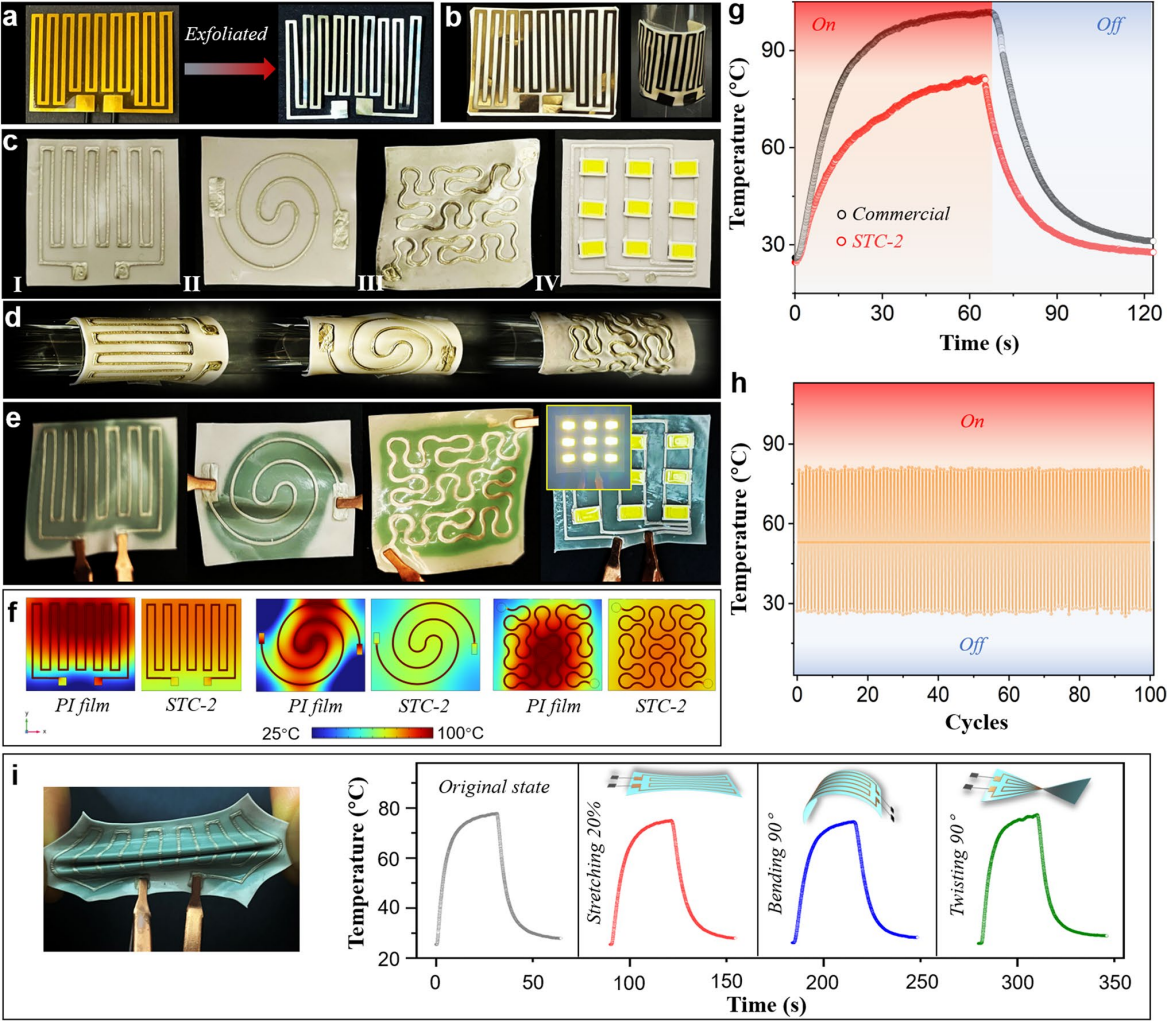
Thermal management application for flexible planar electrodes. **a** Photos of a commercial Kirigami electrode on a PI substrate. **b** Photos of the Kirigami electrode self-adhered on the STC-2 membrane with excellent flexibility. **c** Photos of STC-2 membranes with different 3D-printed patterns and electronics (I: polyline-type pattern, II: coil-type pattern, III: embroidery-type pattern, IV: LED arrays). **d** Photos showing flexibility of various 3D-printed circuits on the STC-2 membrane. **e** Photos of thermochromic conversion by heat dissipation. **f** FE simulation (steady model) of the heat diffusion for various 3D-printed circuits on the PI film and STC-2 membrane. **g** Temperature profile with time. **h** Cycles of the temperature change of the Kirigami electrode on the STC-2 membrane. **i** Demonstration of the steady thermal management and thermochromic performances for patterned circuits under stretching and deformations

The practicability of thermal management for soft electronics is important. It should be emphasized that here, attempts were made on real flexible circuits or patterned electrodes rather than nonflexible LED chips or built-in devices, which are preferentially adopted in most studies [9, 13–17]. For instance, we first exfoliated a commercial Al foil Kirigami electrode from a PI substrate (Figs. 6a and S25a). The Kirigami electrode (thickness: ~ 20 μm) originally attached by glues to the PI substrate was papery and elastic. It was easily folded, wrapped and elongated. Hence, directly attaching such a flexible electrode to an ordinary heat spreader with glue is arduous. However, in our case, the self-attachment of the bare Kirigami electrode on the STC-2 membrane (thickness: ~ 50 μm) was easy (Figs. 6b and S25b). Due to the strong viscidity of STC-2, the device can afford multiple bending and curving and even more elongation (Fig. S25c, d, Video S3). The “chameleon-bionic” smart thermal management was then realized by converting accumulated heat to colour changes, consistent with the IR images for heat diffusion (Fig. S25). Similarly, the generality for various soft electronics was illustrated by 3D-printed circuits and LED arrays (Fig. 6c), with polyline-type, coil-type and embroidery-type patterns directly printed on the STC-2 membrane (thickness: ~ 50 μm). Even more complicated LED array circuits were also feasible to fabricate on the membrane. Strong flexibility was demonstrated by rolling the patterned membranes around the rod (Fig. 6d). There was no peeling-off during wrapping, highlighting the excellent self-adhesiveness. Upon powering on, heat dissipation was discerned through thermochromism (Fig. 6e). Interestingly, we observed rapid thermal diffusion from the circuits to the surroundings via green colour expansion (Videos S4-S6). When the power was off, a fast cooling process was identified within 20 s since the colour gradually turned from green to white. Furthermore, the temperature distribution during heat spreading was simulated. As shown in Fig. 6f, all STC-2-loaded circuits exhibited a relatively homogenous temperature dispersion at approximately 75–80 °C, corresponding to the IR images (Fig. S26a). However, for the PI loaded case, the circuit temperature reached as high as 100 °C, and the temperature distribution became hierarchical. When we set a larger power density for the STC-2 loaded cases, the temperature was still lower than 100 °C and stabilized at approximately 80 °C (Fig. S26b), proving the excellent cooling capacity. The temperature profiles in Fig. S26c revealed rapid cooling (less than 20 s) for all STC-2 loaded cases, in accordance with the fast colour conversion for visible heat dissipation. Then, we disclosed the excellent cooling efficiency from temperature profiles (Fig. 6g), where the STC-2 loaded electrode was cooled rapidly with a temperature of 35 °C within 20 s, less than the 55 °C of the PI substrate loaded case. The excellent sustainability of heat dissipation for STC-2 was revealed by turning the electronics alternately on and off for 100 cycles, as shown in Fig. 6h. In addition, the outstanding thermal sensing property of STC-2 by continuous thermochromism for several cycles was also tested in Fig. S25j, therefore enabling the dynamic recognition of temperature variation in soft electronics. Furthermore, the good heat diffusion capacity integrated with thermochromic performance can be maintained during the stretching, 90° bending or twisting of STC-2 (Figs. 6i and S27), hence confirming the deformability. In addition to soft electronics, STC-2 was also competent as a universal cooling component towards nonflexible devices such as field effect transistors (MOSFETs), LED chips and heatable ceramic plates (Figs. S28-S29).

## Conclusions

To the best of our knowledge, a thermochromic and viscoelastic nanocomposite with a nacre-like structure integrating self-adhesive and self-healable characteristics was reported for the first time. The VAF-derived membrane consisting of W-SPU, BNNS and TCM exhibited outstanding mechanical and thermal properties, *i.e.*, impressive stretchability, considerable viscoelasticity and remarkable thermoconductivity. Intriguingly, the introduction of TCM can produce strong interfacial interactions with W-SPU and BNNS, hence not only contributing to thermostimulus sensitivity but also facilitating reinforcement. More importantly, the anti-scraping ability, ultrarobust adhesion and self-repair capacity with the substrate allowed STC-2 to address the gaps of conventional thermoconductive nacre-like composites as heat spreaders. Together with high ҡ_//_, low R_c_, and CTE values, promising foldability and thermal colour-changeability, our nacre-like composite demonstrated the realization of smart thermal management for different soft electronics such as Kirigami electrodes and 3D-printed circuits. It is therefore anticipated that the design concept will make significant contributions to a series of artificial intelligence materials for advanced thermal applications in the future.

### Supplementary Information

Below is the link to the electronic supplementary material.

Supplementary file1 (MP4 1732 KB)

Supplementary file2 (MP4 1870 KB)

Supplementary file3 (MP4 1619 KB)

Supplementary file4 (MP4 1129 KB)

Supplementary file5 (MP4 1809 KB)

Supplementary file6 (MP4 872 KB)

Supplementary file7 (MP4 2112 KB)

Supplementary file8 (PDF 3100 KB)
