# Supplementary material for: A Thermochromic, Viscoelastic Nacre-like Nanocomposite for the Smart Thermal Management of Planar Electronics
Source: Nanomicro Lett. 2023 Jul 5;15:170. doi: 10.1007/s40820-023-01149-8 (PMC10322808; doi:10.1007/s40820-023-01149-8)
Supplement: Supplementary file 8 — Supplementary file8 (PDF 3100 KB) [file 40820_2023_1149_MOESM8_ESM.pdf]

A Tthermochromic, Viscoelastic Nacre-Like Nanocomposite for the Smart Thermal Management of Planar Electronics

Jiemin Wang[1], Tairan Yang[2], Zequn Wang[3], Xuhui Sun[3], Meng An[3,*], Dan Liu[2,*], Changsheng Zhao[4,*], Gang Zhang[5,*], Weiwei Lei[2,*]

[1]College of Biomedical Engineering, Sichuan University, Chengdu, 610064, P. R. China

[2]Institute for Frontier Materials, Deakin University, Waurn Ponds Campus, Locked Bag 20000, Victoria 3220, Australia

[3]College of Mechanical and Electrical Engineering, Shaanxi University of Science and Technology, Xi'an 710021, P. R. China

[4]College of Polymer Science and Engineering, State Key Laboratory of Polymer Materials Engineering, Sichuan University, Chengdu, 610065, P. R. China

[5]Institute of High Performance Computing A*STAR, Singapore, 138632 Singapore

*Corresponding authors. E-mail: anmeng@sust.edu.cn (Meng An), dan.liu@deakin.edu.au (Dan Liu), zhaochsh70@scu.edu.cn (Changsheng Zhao), zhangg@ihpc.a-star.edu.sg (Gang Zhang), weiwei.lei@deakin.edu.au (Weiwei Lei)

S1 Computational Methodology

S1.1 Structural Models

All-atom MD simulations were performed to calculate the interfacial thermal resistances between Al thin films and three types of thermal interfacial materials i.e., epoxy paste, polyurethane with disulfide bond with thermochromic molecules (W-SPU/TCM), and STC-2. The epoxy, W-SPU, TCM, and boron nitride nanosheets (BNNS) [S1] were placed together in a simulation cell with a dimension of 5 × 5 × 8 nm^3 and the relaxation process performed according to the method previously described in literature [S2]. The CVFF force-field parameters and partial atomic charges are adopted to describe the interatomic interactions among the epoxy, W-SPU, TCM and BNNS molecules. Periodic boundary conditions in all three dimensions were applied. The cutoff radius for long-range energy calculations was set to be 12 Å. The contribution of long-range interactions was calculated via the particle-particle-particle-mesh solver. The Newton's equations of motion were time-integrated with a time-step of 0.1 fs using Large-scale Atomic/Molecular Massively Parallel Simulation package developed by Sandia National Laboratories [S3]. The Visual Molecular Dynamics was used to visualize the trajectories generated during MD simulations [S4].

S1.2 Interfacial Thermal Resistance Simulations

The interfacial thermal transport between Al thin film and thermal interface material

are calculated utilizing molecular dynamics simulations of a transient heating technique. This method mimics the short-pulse laser excitation in experiment and has been successfully predict the thermal resistance at the interfaces of metals/amorphous materials [S5]. Initially, the interface systems are relaxed under NPT, NVT and NVE ensembles for 0.5 ns, respectively. After full equilibration at 300 K, an ultrafast heat pulse is added to Al thin film for 50 fs to raise the temperature to 480 K while the temperature of the polymers remained unchanged. Then the system is relaxed to reach a new steady state when heat energy is transported from the hot source to the cold reservoir across the interface. The total energies of Al thin film and polymer systems are recorded at every time step for 50 ps, respectively. In the process of heat conduction at interfaces, the exchanged heat flux through the interface is correlated with the energy variations in polymers due to the whole system in a vacuum, which can be written as:

$$\frac{\partial E_t}{\partial t} = \frac{A(T_{Al} - T_{TIM})}{R} \quad \text{(S1)}$$

where E_t is the total energy of the polymer systems at time t, A is the interface contact area between Al thin films and polymer systems, T_{Al} and T_{TIM} are the temperatures of the Al thin film and thermal interface material systems, respectively. The evolutions of temperature and total energy after 50 fs of heat impulse are shown in Fig. S1. By integrating with respect to time t, the equation can be transformed as:

$$E_t = E_0 + \frac{A}{R}\int_0^t (T_{Al} - T_{TIMs})\, dt \quad \text{(S2)}$$

where E_0 is the initial energy of the TIMs layer. To suppress the noise, the data collected is averaged over every 50-time steps. All the results in this work are ensemble averaged over six independent calculations with different initial conditions.

S1.3 Adsorption Energy Calculation

The two interface models of BNNS/W-SPU and BNNS/TCM/W-SPU interfaces were constructed, where the dimension of the cell box was 50 Å×50 Å×53 Å with vaccum space of 50 Å. The universal force field was adopted to describe the interatomic interactions of interface systems. The energy minimization and relaxation optimization were performed under NPT ensemble of a constant temperature (600 K) and volume within 500 ps. All the molecules are non-fixed to compute the authentic situation. Then the adsorption energy was calculated accordingly.

S2 Supplementary Figures and Tables

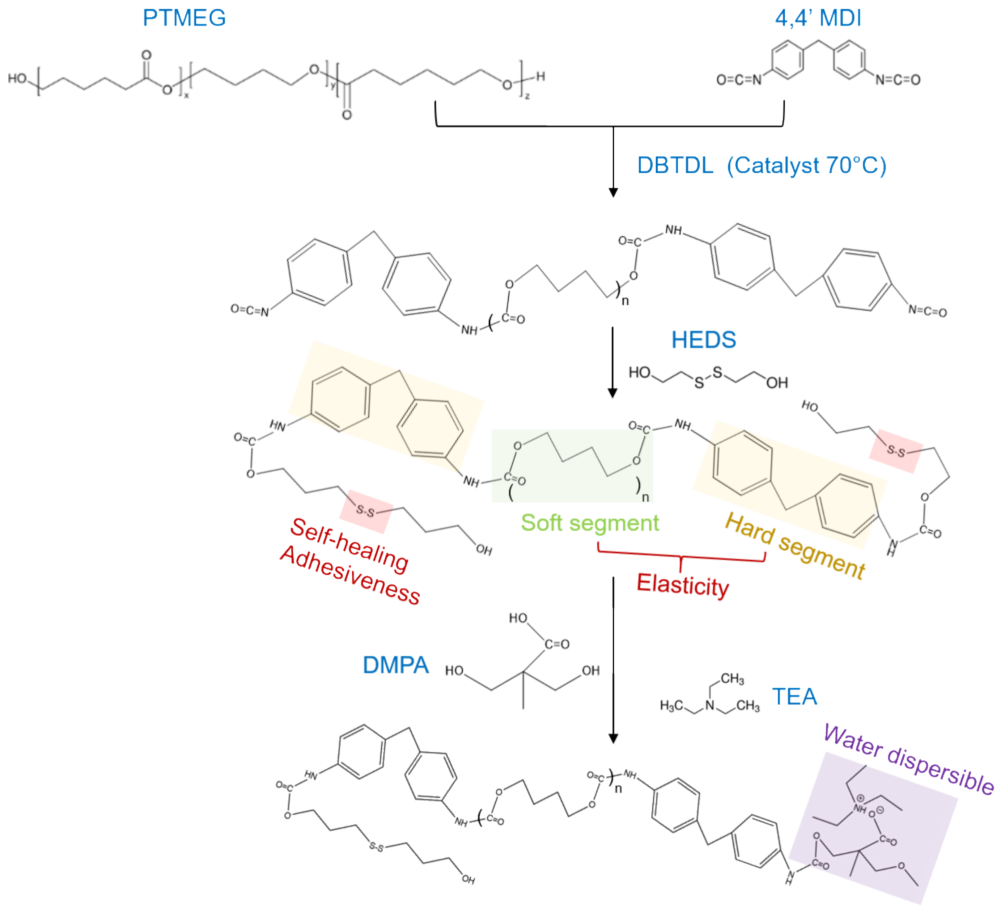

Fig. S1 The scheme of synthesis of W-SPU

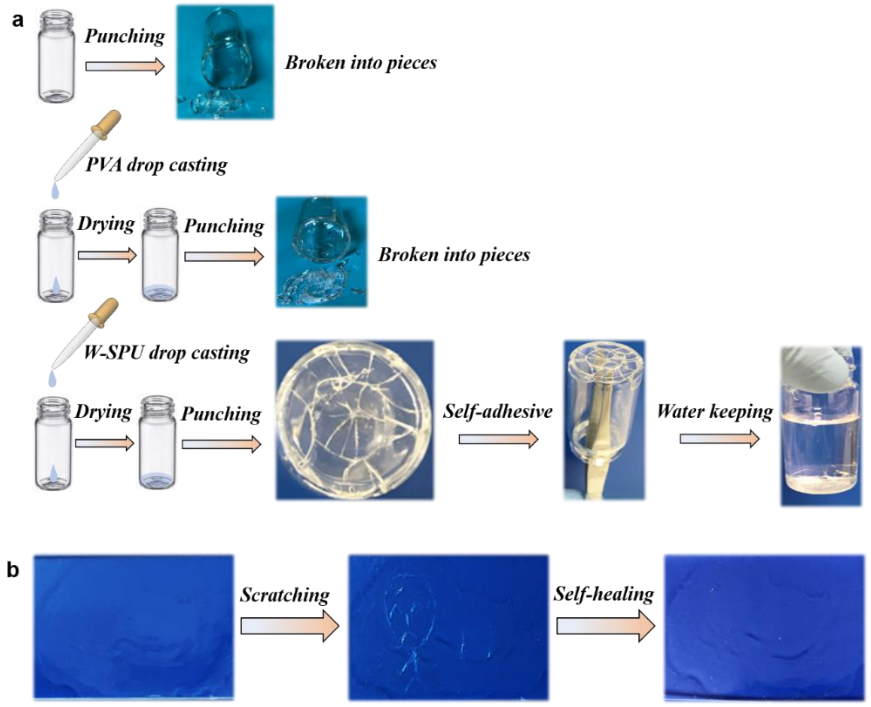

Fig. S2 (**a**) The outstanding self-adhesiveness of W-SPU as the coating membrane for glass vial protection. (**b**) The excellent self-healing properties of W-SPU after scratching

Here, the blank glass vial, polyvinyl alcohol (PVA) solution drop-casted vial and W-SPU solution drop-casted vial were employed to verify the self-adhesiveness. Obviously, the W-SPU coated case was able to stand punching, leaving the bottom of vial integrated rather than being broken into pieces. Hence, it fully highlighted the outstanding self-adhesiveness that was promising for interfacial materials.

In addition, the W-SPU also showed good self-healing properties after deep scratching. It can be seen that, after self-healing, the transparency remained no changes.

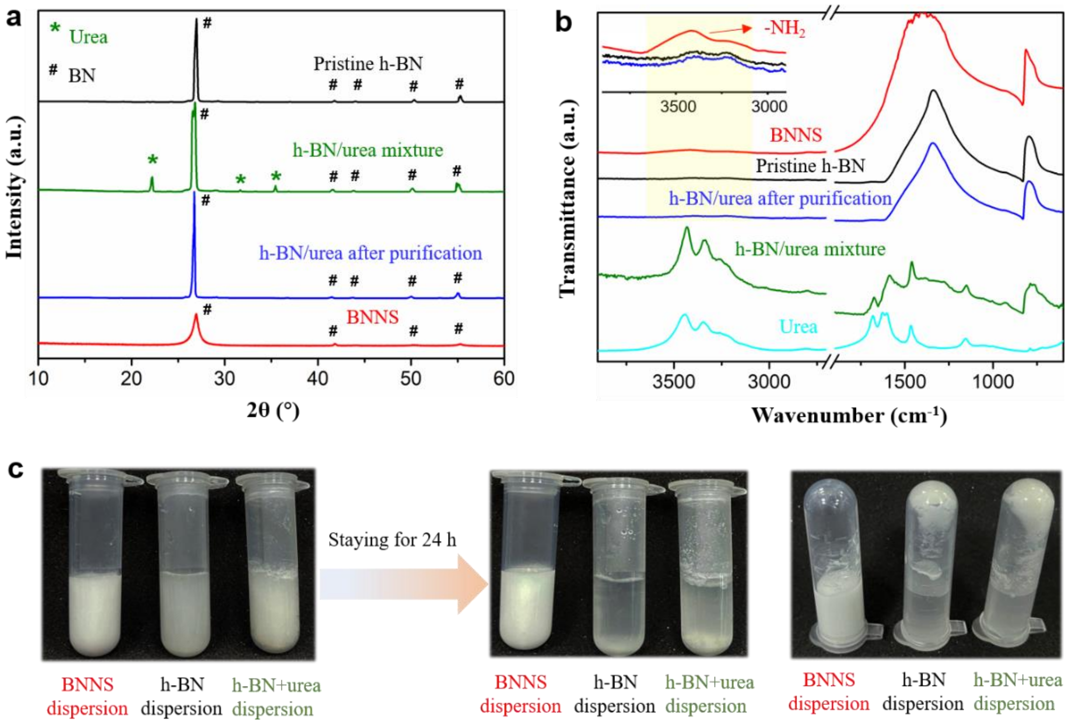

Fig. S3 (**a**) The XRD patterns and (**b**) The FTIR spectra for each case: pristine h-BN, h-BN/urea mixture and BNNS. (**c**) The photos showing the aqueous colloid stability for each case. Note: the purification of h-BN/urea is the process to eliminate the urea molecules from h-BN/urea mixture through continuous dialysis for 1 week in de-ionized water.

It can be found that only the XRD pattern of BNNS showed dramatically broadened and less intensive peak (002) due to the size reduction and layer exfoliation,[1] whereas others remained the pointed peak (Fig. S3a). For h-BN/urea mixture, there were both urea and h-BN characteristic peaks existing. However, after purification, namely removing urea molecules, the XRD pattern of the mixture sample retained no change from pristine h-BN. Similarly, in the FTIR spectra (Fig. S3b), we can observe the B-N stretching and bending characteristic bands at ~1300 cm^{-1} and ~850 cm^{-1} for all BN samples [S1]. However, only primary amine groups stretching (3500-3300 cm^{-1}) existed in the spectrum of BNNS, which was not detected in both spectra of pristine h-BN and h-BN/urea mixture after purification. The appearance of N–H stretching suggested that the occurrence of $-NH_2$ functionalization in BNNS. In addition, from Fig. S3c, BNNS aqueous colloid was stable while both pristine h-BN and h-BN/urea mixture dispersions

appeared to precipitate after 24 h staying. The result is hence a reflection of the covalent NH_2 species rather than physically mixed urea that yielded hydrophilicity for ready dispersibility in water.

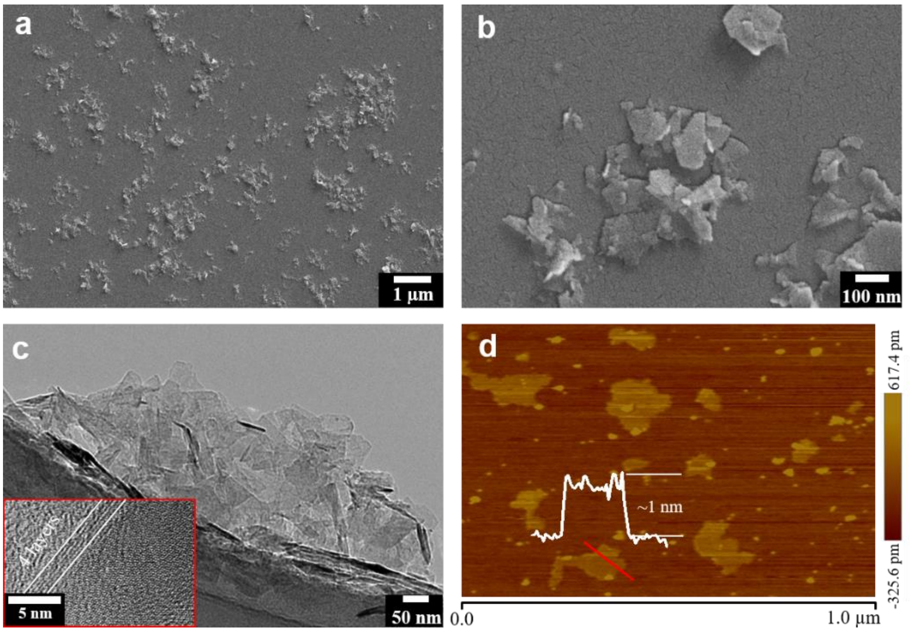

Fig. S4 (**a**) and (**b**) SEM images at different magnifications of BNNS. (**c**) TEM image of BNNS (The inset showing the HRTEM image of BNNS). (**d**) AFM image of BNNS

SEM and TEM images (Fig. S4 a-c) showed the thin and flat laminar feature with average lateral size of 100-300 nm. HRTEM (Fig. S5 c inset) and AFM image (Fig. S4d) further confirmed the thickness of ~1nm with 3-4 monolayers.

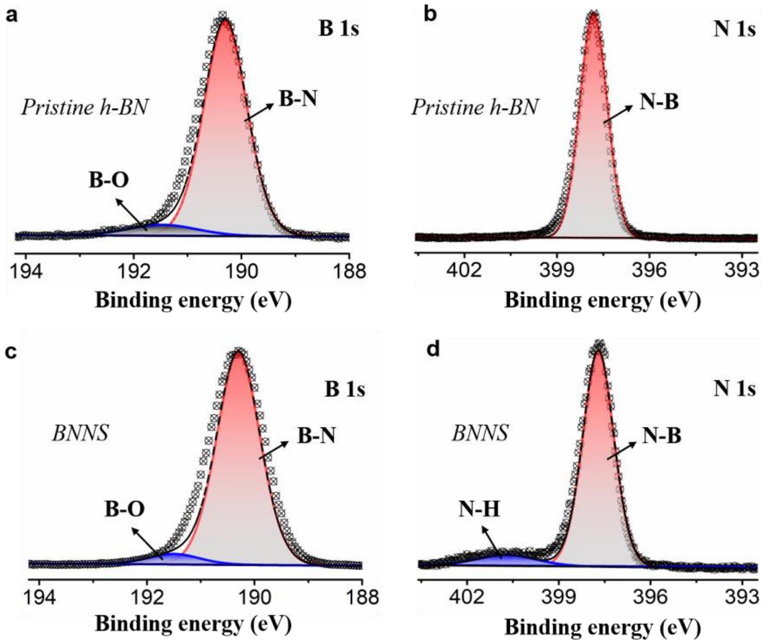

Fig. S5 XPS spectra of pristine h-BN and BNNS. (**a**) B 1s and (**b**) N 1s of pristine h-BN XPS analysis. (**c**) B 1s and (**d**) N 1s of BNNS XPS analysis

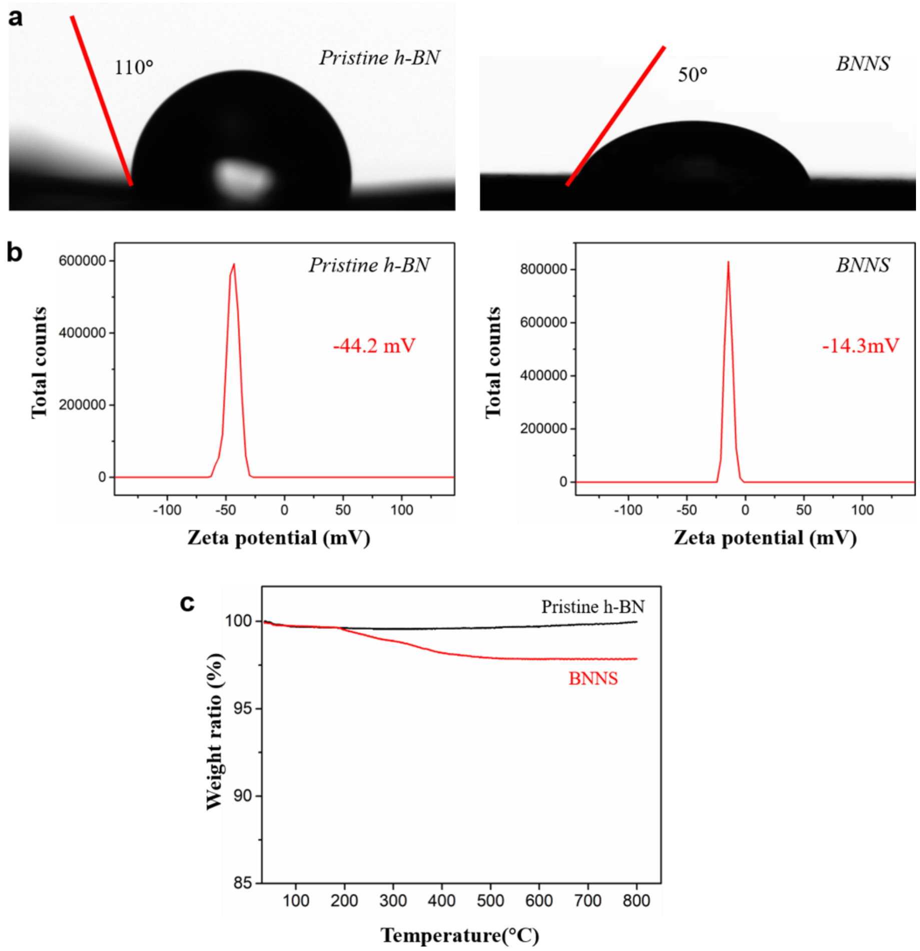

Fig. S6 (**a**) Contact angles, (**b**) Zeta potentials and (**c**) TGA curves for pristine h-BN and BNNS

The prinstine h-BN was hydrophobic with contact angle of 110°. By comparsion, the presence of amino group in BNNS induced a good hydrophilic nature, where the contact angel of BNNS was 50° (Fig. S6a). Besides, we also measured the zeta potentials of prinstine h-BN and BNNS in Figure S6b. The prinstine h-BN possessed zeta potential value of -44.2 mV, due to B-O-H generated on h-BN in water under ultrasonic dispersion [S1] However, for BNNS, the zeta potential shifted to -14.3 mV. The reduced negative zeta potential can be ascribed to the partial neutralization from the $-NH_3^+$ in the amino group functionalized BNNS. Moreover, TGA curve (Fig. S6c) indicated that 2.3 wt% content of loss in BNNS in contrast with prinstine h-BN, which can be asigned to the decomposition of amino functional groups at higher temperature in air.

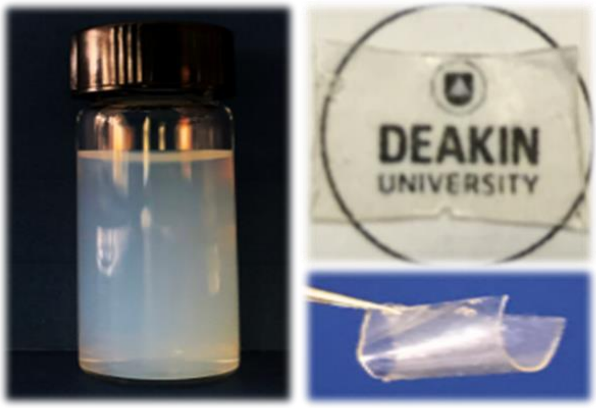

Fig. S7 The optical pictures of the W-SPU water dispersion and the as-obtained transparent W-SPU membranes

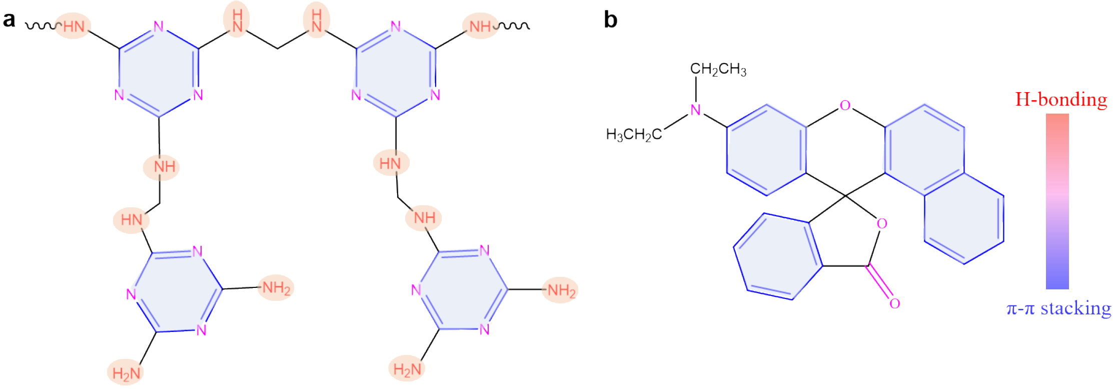

Fig. S8 Main molecular moieties of thermochromic moieties (TCM). (a) urea-formaldehyde resin, (b) 6'-diethylamino-1',2'-benzofluoran

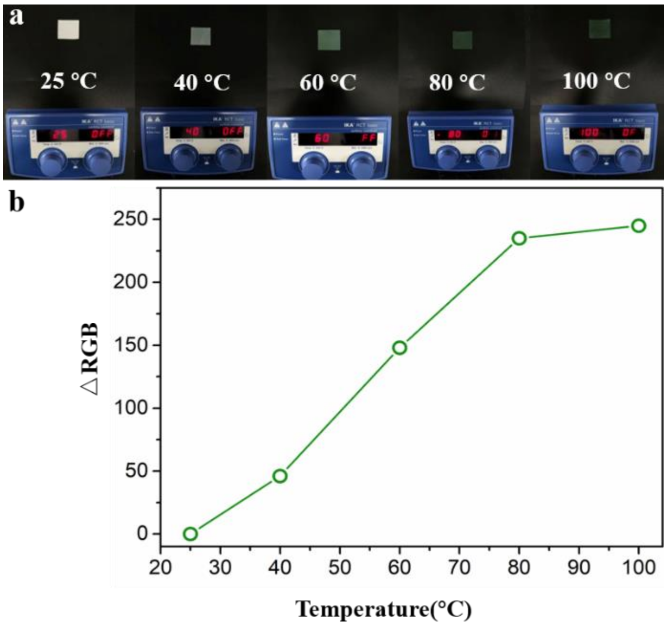

Fig. S9 (**a**) The photos of color shift with temperatures. (**b**) The color change values (ΔRGB) of the STC-2 membrane

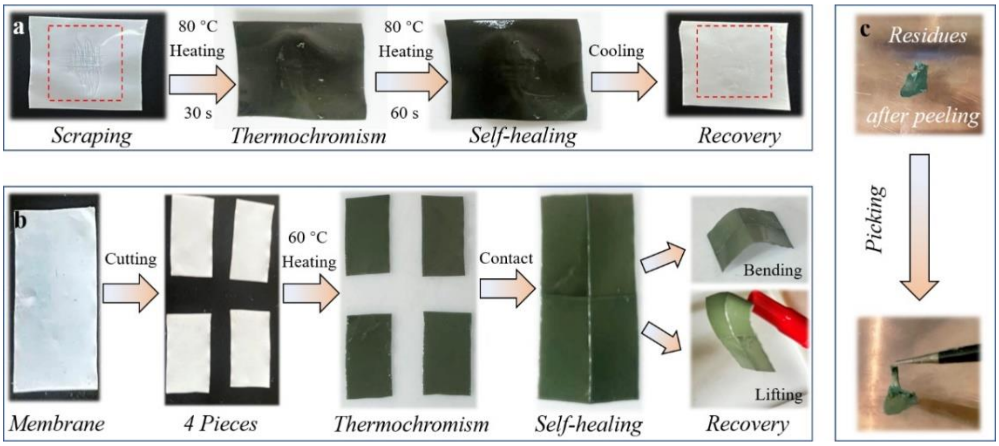

Fig. S10 The photos showing the thermochromic performance during (**a**) scraping and recovery processes, (**b**) fracture and recovery processes. (**c**) The residues after membrane peeled from Cu substrate

From Fig. 10a-c, it can be observed that, despite scraping and fracture, the membrane still remained excellent thermochromism property without appearing non-uniformity in the color. After recovery, the property was keeping unchanged no matter what pre-treatment it suffered.

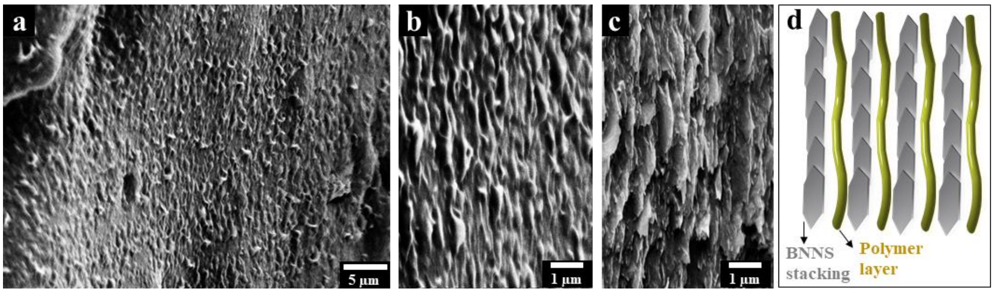

Fig. S11 (**a, b**) SEM image of cross-section of STC-2 with low and high magnification, (**c**) SEM image cross-section of STC-2 after TGA removing the organic phase, (**d**) Schematic illustration to show the BNNS layered stacking in the polymer matrix

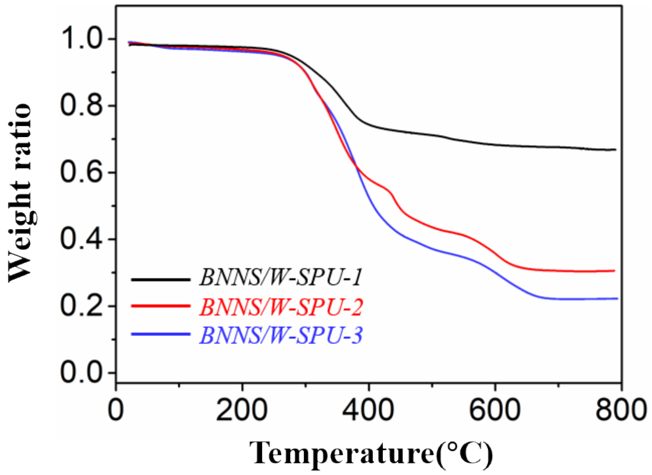

Fig. S12 TGA curves of different BNNS/W-SPU

The weight fractions were quantitatively confirmed by thermogravimetric analysis (TGA). It can be seen that the BNNS contents in the BNNS/W-SPU-1, BNNS/W-SPU-2 and BNNS/W-SPU-3 were ~20, 35 and 70 wt%. We then used them to fabricate STC-1, STC-2 and STC-3 by adding TCM accordingly, and fixed TCM content, naming them as STC-1(~15-18 wt% BNNS), STC-2 (~30-32% wt BNNS) and STC-3 (~63-65 wt% BNNS).

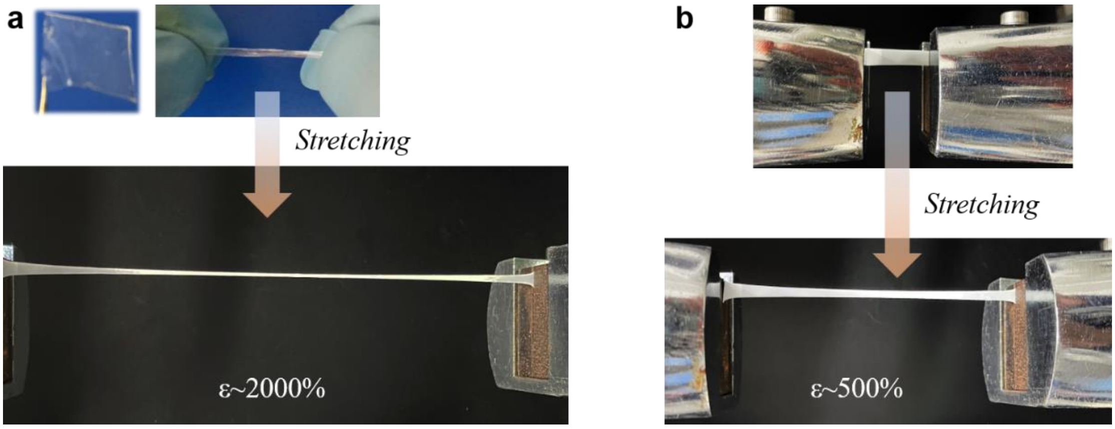

Fig. S13 The optical pictures of (**a**) W-SPU and (**b**) STC-2 membranes under stretching

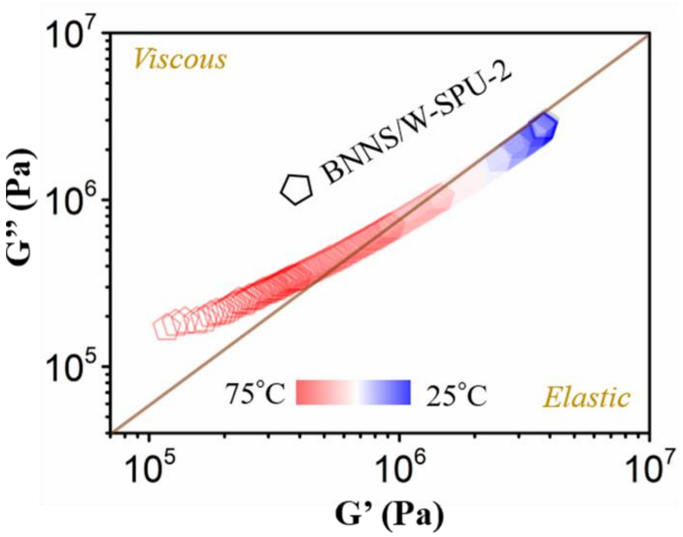

Fig. S14 DMA curves of BNNS/W-SPU-2

Interestingly, when the temperature rose to above 60 °C， BNNS/W-SPU-2 delivered a more distinct viscous state than W-SPU-2 and STC-2. We ascribed it to the lubricating property of BN nanosheets. When the system dynamically became soften, the BNNS played the role of lubricant rather than the physical crosslinking point. However, the existence of TCM with supramolecular interactions can effectively adsorb the BNNS and polymer chain segments, hence locking the fluidity and resulted in a certain solidity.

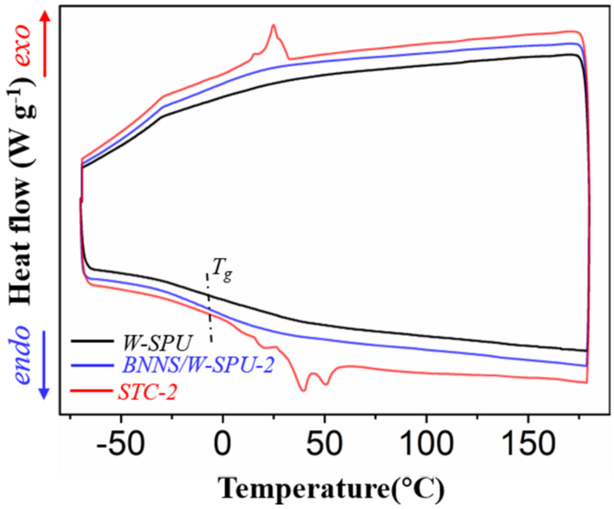

Fig. S15 DSC curves of W-SPU, BNNS/W-SPU-2 and STC-2

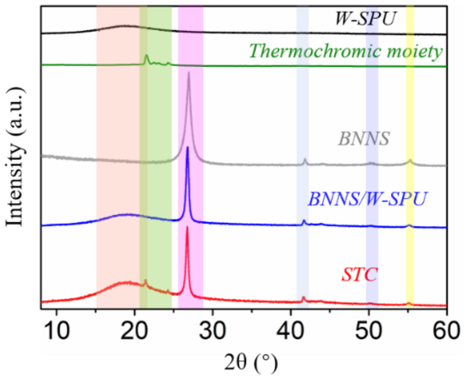

2θ (°)	Assignment
15~20	Semi-crystallite/amorphous area of W-SPU
21~24	Crystallite peaks of urea-formaldehyde resin
26~27	*(002)* peak of BN
41-43	*(100)* peak of BN
51-53	*(102)* peak of BN
55-56	*(004)* peak of BN

Fig. S16 XRD patterns of W-SPU, thermochromic contents, BNNS and STC

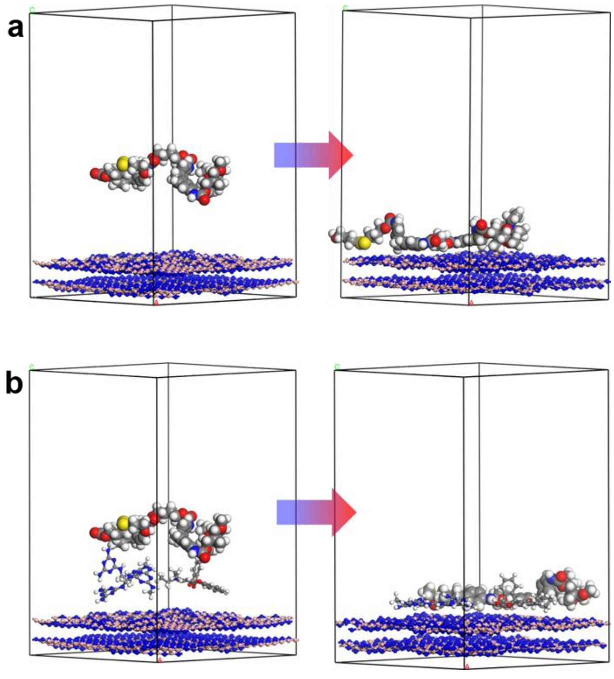

Fig. S17 The molecular adsorption energy models of (**a**) W-SPU/BNNS and (**b**) STC

The adsorption energies were calculated according to the equation below:

$$E_{(Ads)} = E_{(W\text{-}SPU)} + E_{(filler)} - E_{(total)},$$

$E_{(Ads)}$ represented the adsorption energy between W-SPU and filler; $E_{(W\text{-}SPU)}+E_{(filler)}$ were the corresponding energy of W-SPU and filler in the optimized conformation, and $E_{(total)}$ is the total energy of the system.

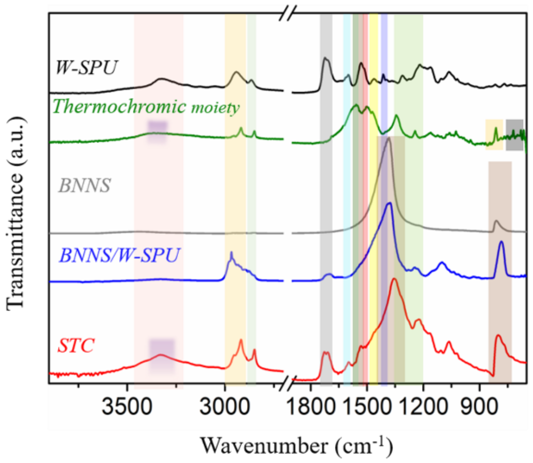

Wavenumber (cm^{-1})	Assignment
3500~3000	H-bond υ (NH/OH)
3300~3000	H-bond υ (C=CH)
2950~2900	υ_a (CH_2)
2880~2830	υ_s (CH_2)
1730~1715	Free υ (C=O) urethane amide I
1670~1640	H-bond υ (C=O) urethane amide I
1650~1620	H-bond γ (N-H) cyanamide
1540~1520	H-bond υ (C-N)+δ (N-H) amide II
1540~1510	υ (C=N) triazine
1480~1430	Free υ (C-N)+ δ (N-H) amide II
1380~1350	Ω(C-H) in CH_2
1400~1300	υ (B-N)
1250~1220	υ (C-N) + δ (N-H) amide III
850~800	γ (B-N-B)
820~800	δ(C=N) triazine
800~650	γ (C-H) aromatic ring

Fig. S18 FTIR of XRD patterns of W-SPU, thermochromic contents, BNNS and STC

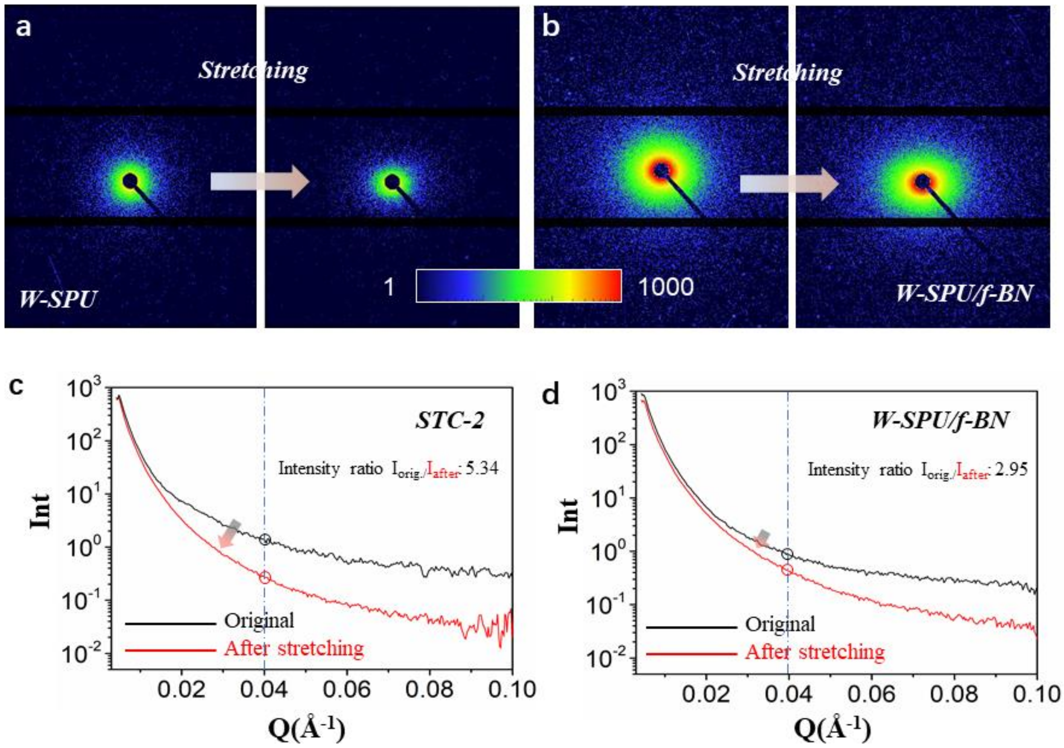

Fig. S19 SAXS of **a**) Original W-SPU and **b**) BNNS/W-SPU-2. SAXS profile of **c**) STC-2 and BNNS/W-SPU-2

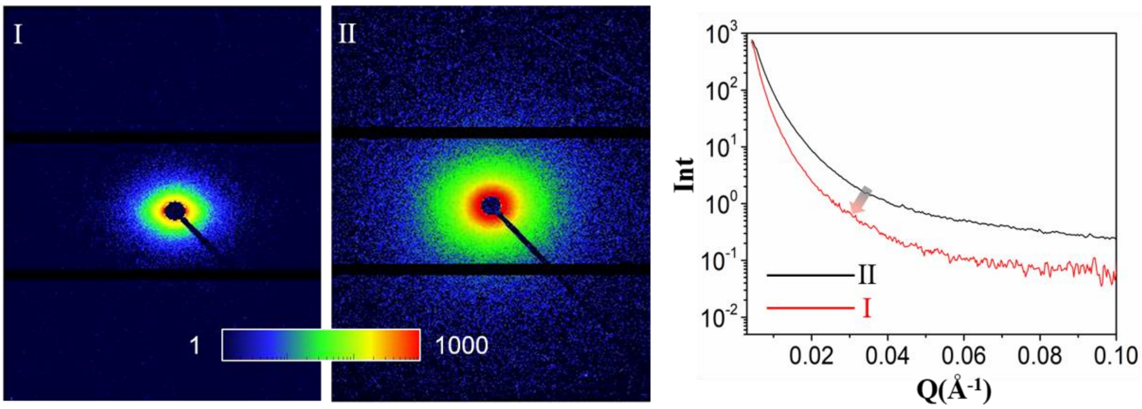

Fig. S20 SAXS pattern and profile of areas I and II

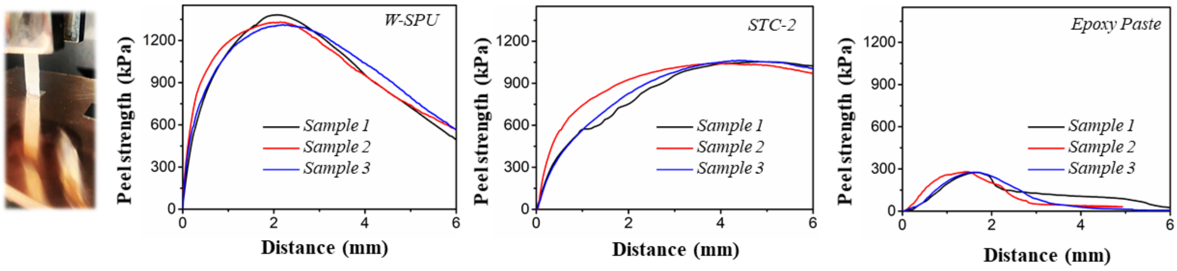

Fig. S21 The adhesive forces measured for different samples with Cu substrate

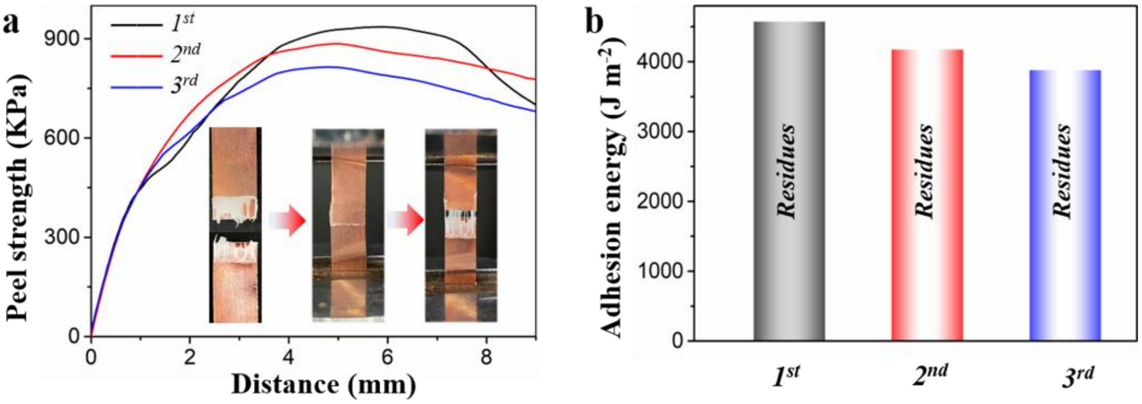

Fig. S22 (**a**) The adhesive forces for residues within 3 times. (**b**) The corresponding adhesion energy

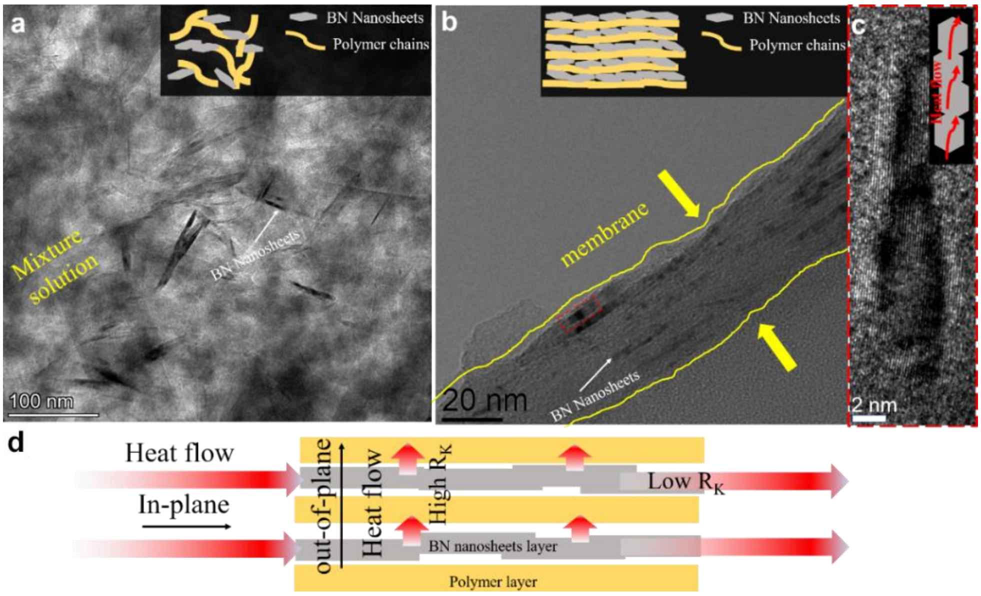

Fig. S23 TEM images of (**a**) Mixture solution of STC-2 and (**b**) Cross-section of the STC-2 membrane. (**c**) HRTEM images of BNNS stacking in the polymer matrix (The magnified area in (**b**). (**d**) The schematic illustration of the heat transfer path in the STC-2 membrane

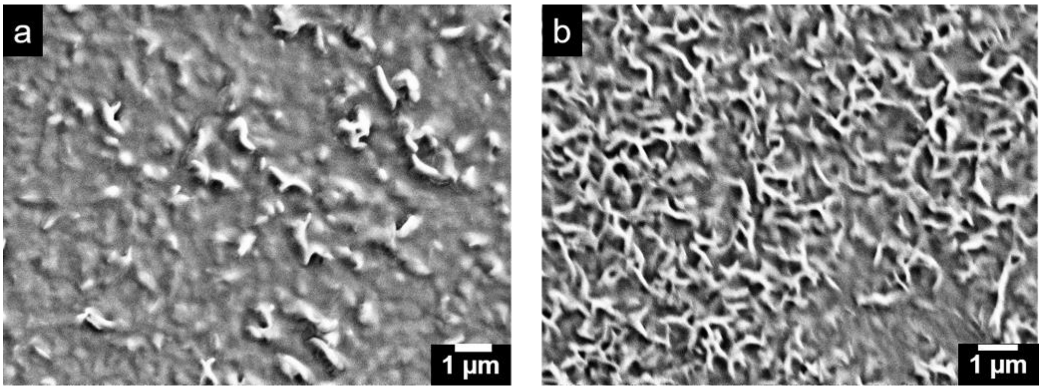

Fig. S24 SEM images of surface area of (**a**) STC-1 and (**b**) STC-3

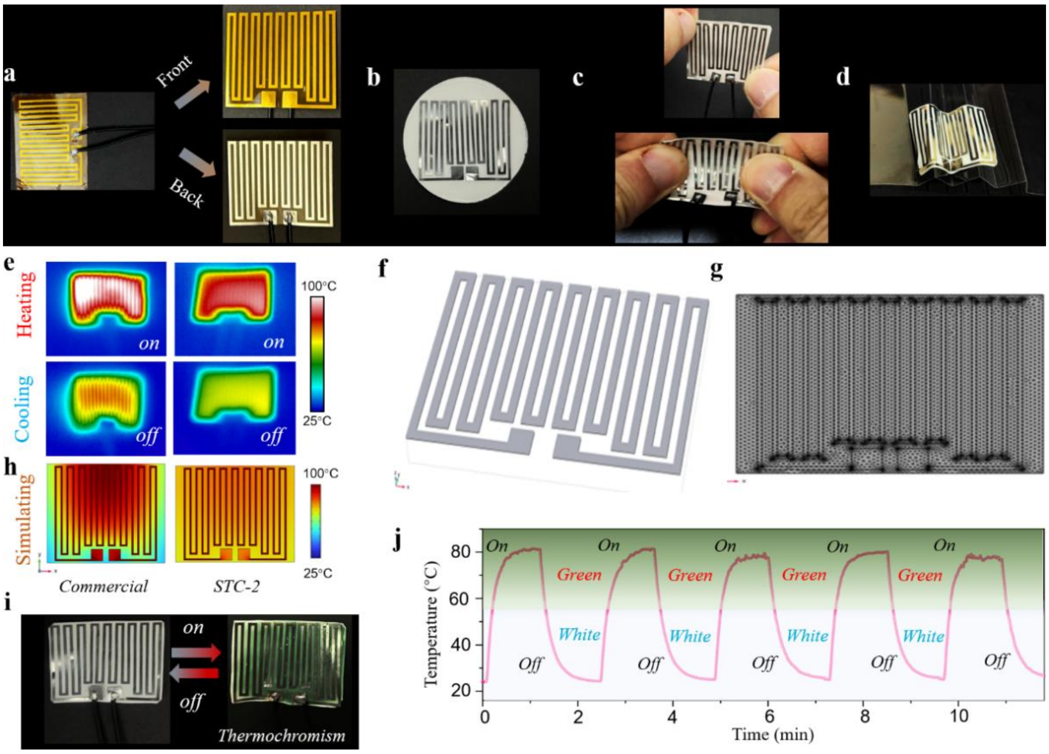

Fig. S25 Photos of (**a**) commercial aluminium (Al) foil Kirigami electrode on polyimide (PI) substrate, (**b**) Kirigami electrode self-adhered on STC-2 membrane and (**c**) Kirigami electrode self-adhered on STC-2 membrane under stretching, (**d**) Kirigami electrode for folding, (**e**) IR images for thermal management comparison of Kirigami electrode. (**f**) The constructed three-dimensional (3D) models of topological electrodes. (**g**) The high-quality meshes of 3D models in the calculation of finite element modelling, (**h**) FE simulation (steady model), (**i**) Photos of thermochromic conversion by heat dissipation, (**j**)Temperature profile of Kirigami electrode on STC-2 with the time: intelligent sensing of the temperature changes from the colour switch by heat dissipation

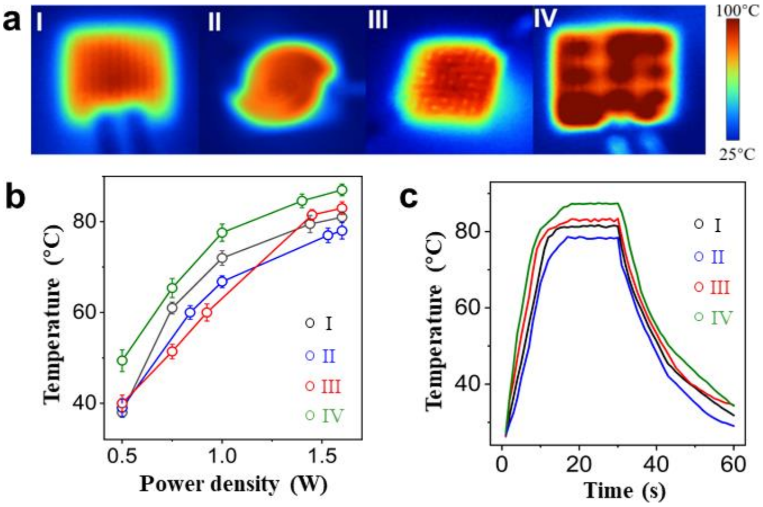

Fig. S26 (**a**) IR images of STC-2 loaded 3D-printed flexible electronics: I: polyline-type pattern, II: coil-type pattern, III: embroidery-type pattern, IV: LED arrays, (**b**) Temperature variation with the applied power density, (**c**) Temperature profile with the time

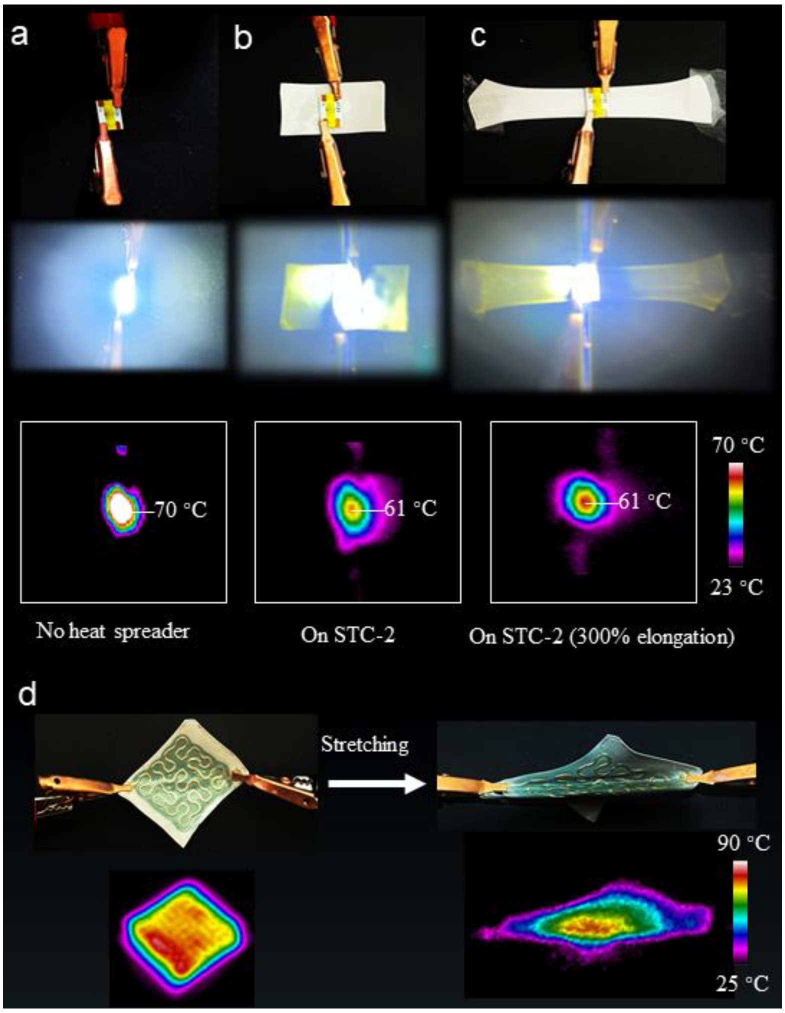

Fig. S27 (**a-c**) Thermal management of LED chip (Photos and high-resolution IR image), Without heat spreader (**a**), Self-adhered on STC-2 membrane (**b**) and Self-adhered on STC-2 membrane (**c**), followed by 300% elongation. (**d**) Thermal management of embroidery-type patterned electrode on STC-2 membrane by stretching (Photos and high-resolution IR image)

From Fig. S27a-c, it can be seen that, by applying STC-2 as self-adhesive heat spreader for LED chip (point heat source), the maximum temperature was decreased by nearly 10 °C. Interestingly, from high-resolution IR image (Fig. S27b-c), the round periphery of temperature distribution for STC-2 membrane was transversely changed a little, rationally ascribing to the orientation by large elongation. Moreover, to reveal the heat dissipating performance for patterned electrode (planar heat source) after elongation, we introduced embroidery-type patterned electrode on STC-2 membrane. As shown in Fig. S27d, the maximum temperature was nearly the same for original and stretched cases, however, the temperature distribution was changed from uniformity to hierarchy. It is reasonable that, during this process, the electrode was also elongated and the heating spot was focused on the center to form point heat source. Thus, the heat spreading mode was from center to the surrounding, resulted in the thermal color hierarchy.

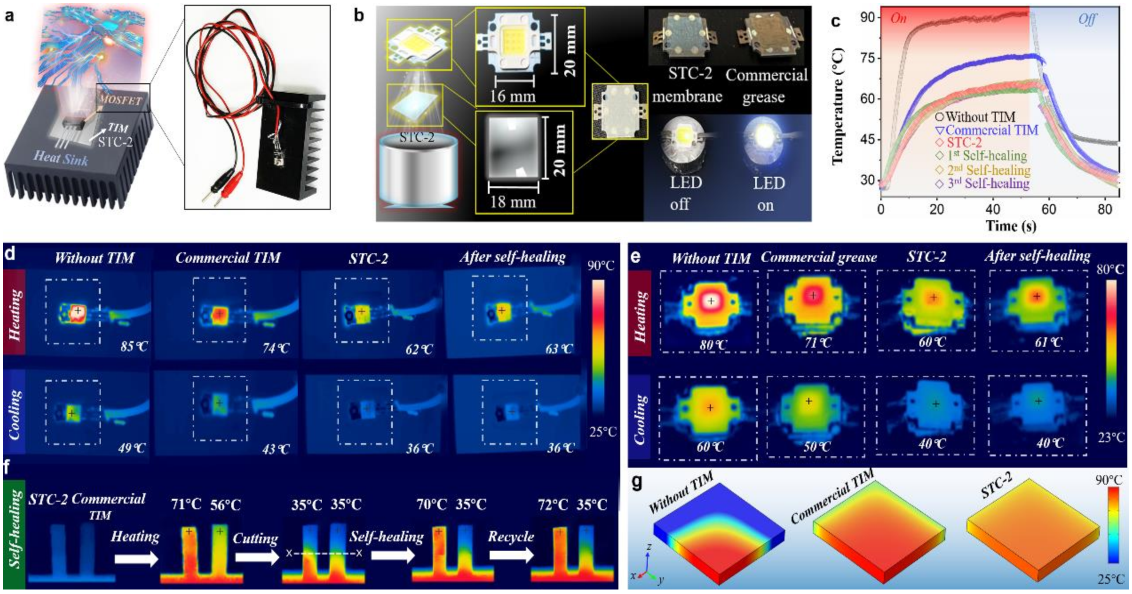

Fig. S28 (**a**) Schematic image and photo of the heat dissipation for MOSFET, (**b**) Schematic images and photos of the heat dissipation for LED chip, (**c**) Temperature distribution against the time for MOSFET with various TIMs, (**d**) IR images depicting the heating and cooling processes for MOSFET, (**e**) IR images depicting the heating and cooling processes for LED chip, (**f**) IR images depicting the self-healing process, (**g**) Finite element simulation (steady model) of the heat diffusion situation in the module among "without TIM", "commercial TIM" and "STC-2".

Applications of the STC-2 membrane as self-adhesive TIM for real MOSFET and LED chip were shown in Fig. S28a-b. It can be seen that, compared with non-TIM and commercial TIM case, the STC-2 loaded cases showed drastic temperature drop even after self-healing for 3 times (Fig. S28c). The effective cooling performances for MOSFET and LED chip were also recorded by IR images in Fig. S28d-e. IR images in Fig. S28f also recorded the recovery of STC-2 for heat dissipation after self-healing, which commercial TIM cannot afford. The superior heat spreading performance was also verified by FE simulation (Fig. S28g).

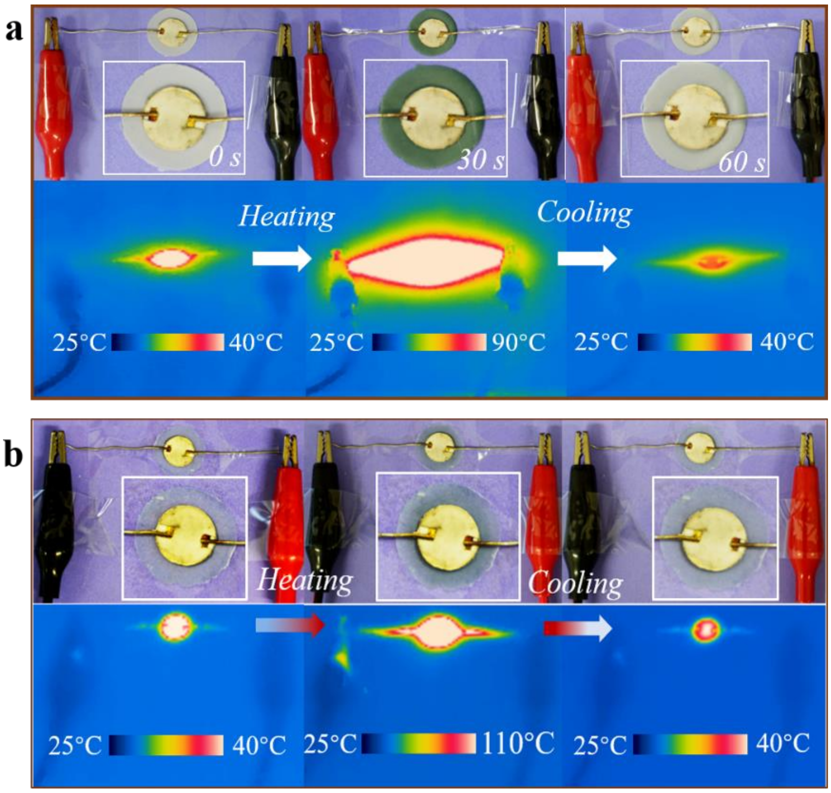

Fig. S29 (a) Synchronous photos and IR images of STC-2 membrane for color-changeable heat dissipation of ceramic heater. (**b**) Synchronous photos and IR images of composite membrane without BNNS for color-changeable heat dissipation of devices

Excellent cooling capacity integrated with thermochromic performances for STC-2 membrane as thermal spreader can be also demonstrated by comparing the two membranes in Fig. S29a-b.

Table S1 The molecular adsorption energy of models a and b

Molecular model	$E_{(W\text{-}SPU)}$ /kcal mol^{-1}	$E_{(Filler)}$ /kcal mol^{-1}	$E_{(Total)}$ /kcal mol^{-1}	$E_{(Ads)}$ /kcal mol^{-1}	$E_{(Ads)}$ /kJ mol^{-1}
**a**	191.66543	-51864.872526	-51776.92076	103.713664	434.13002
**b**	147.053146	-52039.707015	-52078.96997	186.3161	779.891586

Table S2 The comprehensive comparison of the properties among different thermo-conductive composites with nacre-like layered structure

Samples	$\kappa_{//}$ /(W m^{-1} K^{-1})	Elongation (%)	Adhesion	Self-healing	Insulation	Thermal response	Anti-scraping	Refs.
BNNS/PVA	6.9	4	No	No	Yes	No	No report	S6
BNNS/PVA	35	25	No	No	Yes	No	No report	S7
BNNS/PI	51	12.5	No	No	Yes	No	No report	S8
BNNS/PVDF	16.3	No report	No	No	Yes	No	No report	S9
BNNS/soy protein	2.4	11	No	No	Yes	No	No report	S10
BNNS/CNF	9.1	No report	No	No	Yes	No	No report	S11
BNNS/Kevlar	46.7	5.3	No	No	Yes	No	No report	S12
BNNS/epoxy	6.07	No report	Yes	No	Yes	No	No report	S13
GO/PVA	No report	6	No	No	Yes	No	No report	S14
MXene/PVA/PEDOT	No report	3.96	No	No	No	photothermal	No report	S15
Al/DA polymer	No report	15	No report	80%	No report	Shape-memory	No report	S16
STC-2	~30	500	~4680 J m^{-2},	80-100%	Yes	thermochromic	Yes	Ours

Note: $\kappa_{//}$ represents in-plane thermal conductivity.

Table S3 Detailed dimensions and physical parameters of the model

	size x (mm)	size y (mm)	size z (mm)	width (mm)	k (W/(m·K))
Base material(epoxy resin)	20	17	0.1		4.5
Base material(new)	20	17	0.1		30
Electrode wire(argentum)			0.03	0.3	420
Electrode wire(aluminium)			0.03	0.3	237

Supplementary References

[S1] W. Lei, V. N. Mochalin, D. Liu, S. Qin, Y. Gogotsi et al., Boron nitride colloidal solutions, ultralight aerogels and freestanding membranes through one-step exfoliation and functionalization. Nat. Commun. **2015**, 6, 8849. https://doi.org/10.1038/ncomms9849

[S2] Cheng Chi, Meng An, Xin Qi, Yang Li, Ruihan Zhang, Selectively tuning ionic thermopower in all-solid-state flexible polymer composites for thermal sensing. Nat. Communications **2022**, 13, 221. https://doi.org/10.1038/s41467-021-27885-2

[S3] S. Plimpton, Fast parallel algorithms for short-range molecular dynamics. J. Comput. Phys. **1995**, 117, 1-19. https://doi.org/10.1006/jcph.1995.1039

[S4] W. Humphrey, A. Dalke, Schulten, VMD: Visual molecular dynamics. J. Molecular Graph. **1996**, 14, 33-38. https://doi.org/10.1016/0263-7855(96)00018-5

[S5] X. Wei, T. Zhang, T. Luo, Thermal energy transport across hard–soft interfaces. ACS Energy Lett. **2017**, 2, 2283-2292. https://doi.org/10.1021/acsenergylett.7b00570

[S6] X. Zeng, L. Ye, S. Yu, H. Li, R. Sun et al., Artificial nacre-like papers based on noncovalent functionalized boron nitride nanosheets with excellent mechanical and thermally conductive properties. Nanoscale **2015**, 7, 6774. https://doi.org/10.1039/C5NR00228A

[S7] J. Wang, Y. Wu, Y. Xue, D. Liu, X. Wang et al., Super-compatible functional boron nitride nanosheets/polymer films with excellent mechanical properties and ultra-high thermal conductivity for thermal management. J. Mater. Chem. C **2018**, 6, 1363. https://doi.org/10.1039/C7TC04860B

[S8] J. Wang, D. Liu, Q. Li, C. Chen, Z. Chen et al., Nacre-bionic nanocomposite membrane for efficient in-plane dissipation heat harvest under high temperature. J. Materiomics **2021**, 7, 219. https://doi.org/10.1016/j.jmat.2020.08.006

[S9] J. Chen, X. Huang, B. Sun, P. Jiang, Highly thermally conductive yet electrically insulating polymer/boron nitride nanosheets nanocomposite films for improved thermal management capability. ACS Nano **2019**, 13, 337. https://doi.org/10.1021/acsnano.8b06290

[S10] S. Jiang, Y. Wei, S. Q. Shi, Y. Dong, C. Xia et al., Nacre-inspired strong and multifunctional soy protein-based nanocomposite materials for easy heat-dissipative mobile phone shell. Nano Lett. **2021**, 21, 3254. https://doi.org/10.1021/acs.nanolett.1c00542

[S11] D. Hu, H. Liu, Y. Ding, W. Ma, Synergetic integration of thermal conductivity and flame resistance in nacre-like nanocellulose composites. Carbohydr. Polym. **2021**, 264, 118058. https://doi.org/10.1016/j.carbpol.2021.118058

[S12] K. Wu, J. Wang, D. Liu, C. Lei, D. Liu et al., Highly thermoconductive, thermostable, and super-flexible film by engineering 1d rigid rod-like aramid nanofiber/2d boron nitride nanosheets. Adv. Mater. **2020**, 32, 1906939. https://doi.org/10.1002/adma.201906939

[S13] J. Han, G. Du, W. Gao, H. Bai, An anisotropically high thermal conductive boron nitride/epoxy composite based on nacre-mimetic 3d network. Adv. Funct. Mater. **2019**, 29, 1900412. https://doi.org/10.1002/adfm.201900412

[S14] J. Zhu, H. Zhang, N. A. Kotov, Thermodynamic and structural insights into nanocomposites engineering by comparing two materials assembly techniques for graphene. ACS Nano **2013**, 7, 4818. https://doi.org/10.1021/nn400972t

[S15] L. Wang, N. Li, Y. Zhang, P. Di, M. Li et al., Flexible multiresponse-actuated nacre-like MXene nanocomposite for wearable human-machine interfacing. Matter **2022**, 5, 3417. https://doi.org/10.1016/j.matt.2022.06.052

[S16] G. Du, A. Mao, J. Yu, J. Hou, N. Zhao et al., Nacre-mimetic composite with intrinsic self-healing and shape-programming capability. Nat. Commun. **2019**, 10, 800. https://doi.org/10.1038/s41467-019-08643-x
